# Olive agroforestry shapes rhizosphere microbiome networks associated with annual crops and impacts the biomass production under low-rainfed conditions

**DOI:** 10.3389/fmicb.2022.977797

**Published:** 2022-10-28

**Authors:** Ameni Ben zineb, Karim Barkaoui, Fatma Karray, Najla Mhiri, Sami Sayadi, Ahmed Mliki, Mahmoud Gargouri

**Affiliations:** ^1^Laboratory of Plant Molecular Physiology, Centre of Biotechnology of Borj-Cedria, Hammam-Lif, Tunisia; ^2^CIRAD, UMR ABSys, Montpellier, France; ^3^ABSys, Univ Montpellier, CIHEAM-IAMM, CIRAD, INRAE, Institut Agro, Montpellier, France; ^4^Laboratory of Environmental Bioprocesses, Centre of Biotechnology of Sfax, Sfax, Tunisia; ^5^Biotechnology Program, Center for Sustainable Development, College of Arts and Sciences, Qatar University, Doha, Qatar

**Keywords:** olive agroforestry, sun cropping system, soil-rhizosphere, microbial network keystones, cereals, legumes

## Abstract

Agroforestry (AF) is a promising land-use system to mitigate water deficiency, particularly in semi-arid areas. However, the belowground microbes associated with crops below trees remain seldom addressed. This study aimed at elucidating the effects of olive AF system intercropped with durum wheat (Dw), barely (Ba), chickpea (Cp), or faba bean (Fb) on crops biomass and their soil-rhizosphere microbial networks as compared to conventional full sun cropping (SC) under rainfed conditions. To test the hypothesis, we compared the prokaryotic and the fungal communities inhabiting the rhizosphere of two cereals and legumes grown either in AF or SC. We determined the most suitable annual crop species in AF under low-rainfed conditions. Moreover, to deepen our understanding of the rhizosphere network dynamics of annual crops under AF and SC systems, we characterized the microbial hubs that are most likely responsible for modifying the microbial community structure and the variability of crop biomass of each species. Herein, we found that cereals produced significantly more above-ground biomass than legumes following in descending order: Ba > Dw > Cp > Fb, suggesting that crop species play a significant role in improving soil water use and that cereals are well-suited to rainfed conditions within both types of agrosystems. The type of agrosystem shapes crop microbiomes with the only marginal influence of host selection. However, more relevant was to unveil those crops recruits specific bacterial and fungal taxa from the olive-belowground communities. Of the selected soil physicochemical properties, organic matter was the principal driver in shaping the soil microbial structure in the AF system. The co-occurrence network analyses indicated that the AF system generates higher ecological stability than the SC system under stressful climate conditions. Furthermore, legumes’ rhizosphere microbiome possessed a higher resilient capacity than cereals. We also identified different fungal keystones involved in litter decomposition and drought tolerance within AF systems facing the water-scarce condition and promoting crop production within the SC system. Overall, we showed that AF reduces cereal and legume rhizosphere microbial diversity, enhances network complexity, and leads to more stable beneficial microbial communities, especially in severe drought, thus providing more accurate predictions to preserve soil diversity under unfavorable environmental conditions.

## Introduction

Today’s key challenge in the semi-arid area is achieving high agricultural productivity to meet the growing population food demand threatened by biodiversity loss and soil degradation (FAO, 2017). Healthy soils, i.e., the capacity of soils to function as living ecosystems, are rich in organic matter, allowing a high diversity of soil organisms to ensure soil aeration, nutrient cycling, water retention capacity, and plant-microbial symbiosis (Schröder et al., 2016). In most semi-arid areas, however, particularly in the Mediterranean regions, soils are fragile, and intensive agricultural practices and repeated drought threaten soil health (Del Pozo et al., 2019). In arable agrosystems, soil organic matter has been reduced to low levels, resulting in poor biological activity, fertility loss, and reduced water retention capacity, with detrimental consequences on crop yields. In those fields, degraded soils exacerbate the sensitivity of crops to climate adversity, and crop yield now critically depends on rainfall, which is increasingly variable from year to year (Tafoughalti et al., 2018). Annual crops like cereals and legumes, which hold a central place in the food systems in the Mediterranean basin, are exposed to a chronic water deficit during the flowering and grain filling stages, leading to a “terminal drought stress,” which impact yields of both cereals (Del Pozo et al., 2016) and grain legumes (Farooq et al., 2017). Climate change is expected to be particularly intense in the Mediterranean basin and will increase the water deficit earlier in the crop cycle, causing severe concern about soil health and crop yields.

Agroecological approaches can effectively address these issues by benefiting from restored soil health and biological interactions within agrosystems. For instance, tree-based agroforestry (AF) systems can be more profitable than sun crop (SC) cultivation (without trees), especially under arid conditions due to positive interactions between trees and crops. AF is increasingly recognized as a sustainable way to reconcile agricultural production, soil biodiversity, environmental protection, and climate change mitigation (Guillot et al., 2019; Muchane et al., 2020). The most recognized advantage of AF is the enhancement of the ecosystem services by controlling surface runoff and erosion, increasing soil fertility, improving resilience to extreme weather, and ensuring food security (George et al., 2012; Lorenz and Lal, 2014; Do et al., 2020). Widespread forms of AF located in the semi-arid regions of sub-Saharan Africa and Asia include for instance: millet, cotton, and maize grown in the interspaces between rows of planted trees like acacia (*Acacia* sp.), parkia (*Parkia biglobosa*) and shea (*Vitellaria paradoxa*) in sub-Saharan Africa regions (Ramachandran Nair, 2013); cereals grown in the shade of jujube, apricot and walnut trees in China (Qiao et al., 2019), and under almond trees in Iran (Abbasi Surki et al., 2020a). In Mediterranean regions, olive (*Olea europaea* subsp. *europaea* var. *europaea*) is the most cultivated tree species. Olive has great ecological and socio-economical relevance (Caliz et al., 2015), is one of the least nutrients-demanding grown trees, and provides a considerable amount of shade to other plants and animals (Sofo and Manfreda, 2007). In olive AF, different types of crops may be found: (a) annual crops, i.e., cereals, legumes (b) vegetables or small fruit crops like potatoes, melons, tomatoes, legumes, or (c) cover crops, i.e., spontaneous species or sometimes sown grasses (Daoui and Fatemi, 2014; Razouk et al., 2016; Lauri et al., 2019; Castellano-Hinojosa and Strauss, 2020), which have an absolute advantage on the health and yield of olive orchards.

Indeed, several studies have been focused on the benefits and impacts of cover crops to olive AF and its soil microbiome structure (Moreno et al., 2009; Sofo et al., 2010, 2014; Montes-Borrego et al., 2014). For instance, cover crops increased olive rhizosphere soil’s bacterial and fungal diversity. These changes in microbial diversity were positively related to an increase in soil organic matter content and the decomposition of complex polymers in olive orchards (Sofo et al., 2010, 2014; Montes-Borrego et al., 2014). It has also been reported that these microbial changes can mitigate the negative impact of herbicides on soil microbial diversity and activity (Moreno et al., 2009), which improve soil health and olive yield. The relative importance of archaeal taxa in olive-based agrosystems was also investigated, revealing a positive correlation with soil organic N content and exchangeable potassium (Caliz et al., 2015). However, while cover crops have recognized benefits for the olive microbiome and yield, the effects of olive trees on crops and their microbiome remain largely unknown, especially for cereals and legumes under arid conditions.

Previous studies have acknowledged the effect of trees on the belowground microbial communities associated with annual crops in AF compared to SC. For example, the soil microbial biomass and activity were higher for durum wheat, barley, and pea in walnut-based AF than in SC (Guillot et al., 2019). Similarly, the soil rhizosphere of rapeseed, barley, and bread wheat plants have more abundant bacterial communities in poplar-based AF than in SC (Banerjee et al., 2016). In addition, the relative abundance of N-fixing bacteria of the genera *Bradyrhizobium* and *Mesorhizobium* was promoted by poplar trees in the rhizosphere of barley and maize when grown in AF (Beule et al., 2020; Beule and Karlovsky, 2021). However, despite the advent of molecular tools for high-throughput microbial sequencing, the soil microbiota associated with cereals and legumes in olive-based agrosystems is poorly represented in global microbial databases.

Furthermore, to our knowledge, no comparative field study has simultaneously examined how the abundance, composition, and diversity of the annual crops microbiome is affected by olive trees in AF. Notably, a quantitative evaluation of cereal (e.g., wheat) and legume (e.g., faba bean) crop productivity in olive-based AF has been conducted under contrasting water regimes in Northern Morocco (Temani et al., 2021). This study concluded that AF was more land-productive than SC systems, especially under the driest water regimes. However, the diversity and the structure of the core microbial networks associated with these annual crops in tree-based systems remained unexplored.

Indeed, AF is one of the strategies that may promote greater system sustainability with increasing demand for food. Therefore, it is crucial to improve our holistic understanding of such agroecosystem, specifically the complex interactions between above-and belowground processes, with the increasingly occurring climatic perturbations such as drought. Here, we hypothesized that in the AF system: (i) The presence of olive trees is the primary driver of annual crops’ soil microbial diversity. (ii) The olive tree affects the annual crops’ biomass production as compared to SC system. (iii) That microbial hubs in annual crops’ rhizosphere offer more resilience and adaptability to harsh environmental conditions within AF system.

## Materials and methods

### Study site and climate

The field study was performed in a commercial organic farm located in the Fes-Meknes region in northern Morocco (Latitude: 33.9532132, Longitude: −5.5319484, Elevation: 451 m). The climate is Mediterranean semi-continental, with temperate, rainy winters and warm, dry summers. Mean annual rainfall is 576 mm, and mean annual temperature is 17.2°C (mean minimal temperature = 9.8°C and mean maximal temperature = 25.9°C). However, the year of study (2018–2019) was considerably drier than the long-term average, marked by a severe winter drought episode. Rainfall amount during the crop cycle was only 151 mm, with only 6 mm during winter, about 50% less than what is usually expected (Supplementary Figure S1). This unusual winter drought created harsh growing conditions for rainfed crops, leading most farmers in the region to abandon crops before harvest.

### Experimental design

**Figure 1:**
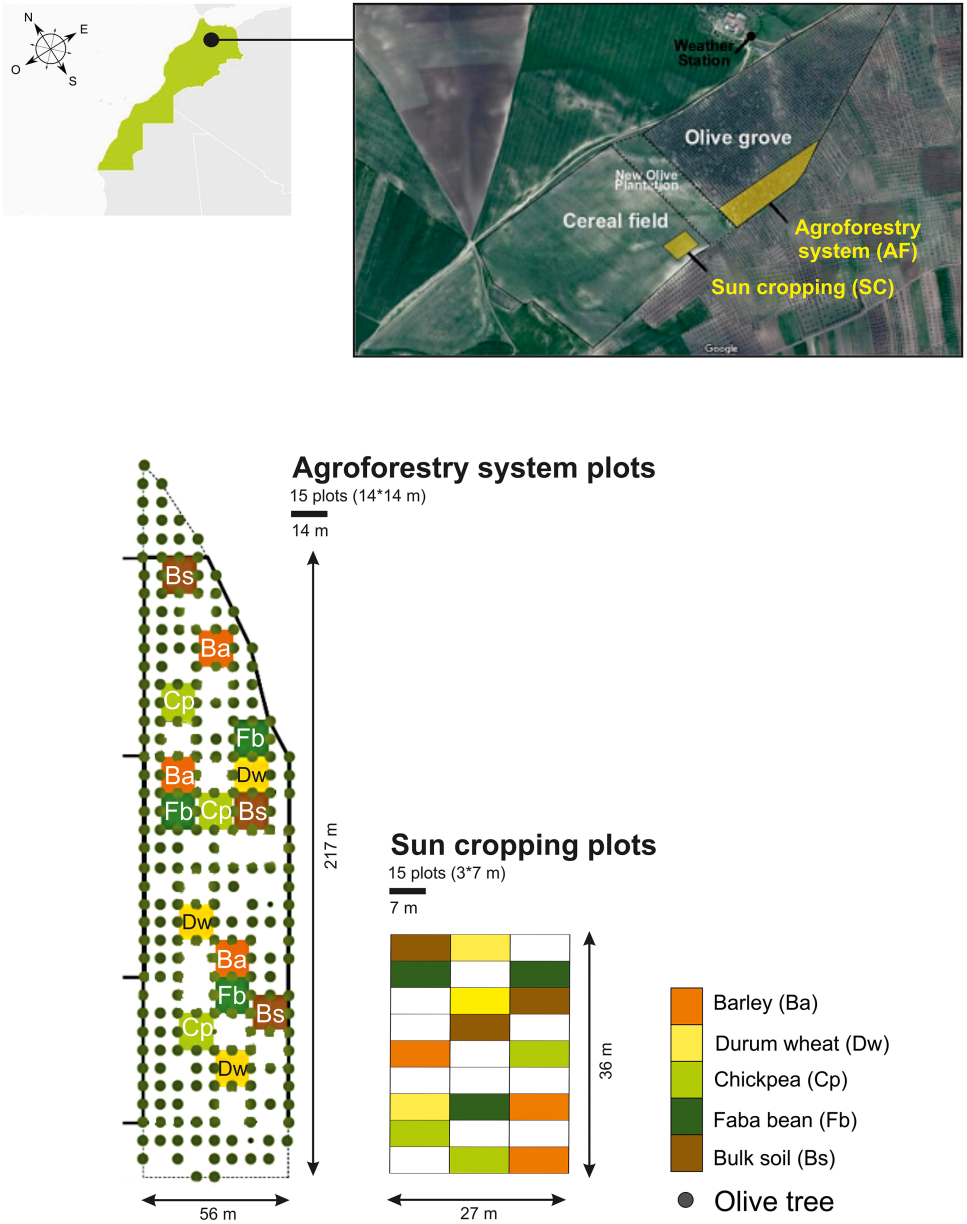
Schematic overview of the experimental design. AF, agroforestry system; SC, full sun cropping; Bs, bulk soil; Ba, barley; Dw, durum wheat; Cp, chickpea; Fb, Faba bean.

We conducted the field experiment over 1 year (from December 2018 to May 2019). We assessed the soil microbiome of two cereals, durum wheat (*Triticum turgidum* subsp*. durum*) and barley (*Hordeum vulgare*), and two legumes, chickpea (*Cicer arietinum*) and faba bean (*Vicia faba*), grown either in full sun cropping (SC) system or in agroforestry (AF) system with olive trees, both under rainfed conditions. In parallel, we also collected bulk soil from each cropping system (control without crops). The SC plots (3 m × 7 m) and AF plots (14 m × 14 m) were sown in two contiguous pieces of land, formerly cultivated as a single plot (annual crops) before olive trees were planted 20 years ago. The experiment had a split-plot design (SC *vs* AF) in which each crop species was replicated three times (three plots in each system) and randomly distributed (Figure 1). The density of olive trees was 200 trees. ha^−1^ with a regular 7 m × 7 m plantation design and they were all of similar size (5 m height; Supplementary Figure S2). Before the experiment and until the year of study, inter-rows were left uncultivated without fertilizer, pesticides, and irrigation. Plots were regularly manually weeded during the vegetative growth.

### Field measurements and sampling of plant and soil

The sampling of the four crops was assessed by randomly selecting 10 plants avoiding borders in each plot at their corresponding flowering stage covering a range between March to April 2019. Each sampled aerial part of plant material was oven-dried to a constant weight at 70°C during 48 h and then weighed to determine plant biomass. Regarding rhizosphere sampling, a soil section depth up to 20 cm until roots of selected plants were found was carefully removed. Rhizosphere soil samples were collected from each plot and pooled (six pooled samples from each AF plot and three pooled samples from each SC plot) for further microbiome analysis (Supplementary Figure S2). Rhizosphere soils were defined as those tightly adhered to the root surface and collected after shaking the roots (Phillips and Fahey, 2006), and the root-free soil was collected as bulk soil. The bulk soil samples were taken from three distant plots of each cropping system’s field where the annual crops were not sown and no plant was grown. Three separate soil cores were obtained at a depth of 0–30 cm for each bulk soil sample with two from both ends and one from the plot’s center. All samples were sifted by a 2 mm sieve to remove all rocks, roots, and large debris. To assess soil physical and chemical characteristics following standardized protocols (Supplementary Data; Table 1), the remaining soil was air-dried, while a part of each soil sample was placed in 15 ml centrifuge tube and stored at −80°C to evaluate the microbial communities. SC and AF bulk soil samples are referred to as BS-SC and BS-AF, respectively, whereas SC and AF rhizospheric soil samples are referred to as Ba-SC and Ba-AF for barely, Dw-SC and Dw-AF for durum wheat, Cp-SC and Cp-AF for chickpeas, Fb-SC and Fb-AF for Faba beans, respectively.

**Table 1:** Soil characteristics.

Treatments		Texture	pH	EC 1/5 (ms cm^−1^)	Total limestone (%)	Active limestone (%)	TOC (%)	N_NH_4_	N_NO_3_	P_2_O_5_	K_2_O	CaO	Na_2_O	MgO	Cl	Cu	Mn	Fe	Zn
								(mg 100 g^−1^)		(mg Kg^−1^)									
AF System	Bs	Clay	8.37	0.17^ab^	20.80	7.36^c^	4.08^a^	0.55	0.55	23.33^ab^	262.3^ab^	16649.6	53.67	304	3.92	0.91^bc^	5.39^a^	7.87	0.52
	Ba	Clay	8.33	0.17^a^	21.30	7.8^c^	4.48^a^	0.52	0.33	19.33^ab^	283.3^ab^	15555.6	54.00	351.33	4.19	1.63^a^	5.51^a^	7.08	0.51
	Dw	Clay	8.33	0.16^bc^	27.67	9.53^bc^	3.78^ab^	0.52	0.27	20.66^ab^	235.6^ab^	15022.6	60.33	272.00	3.39	1.01^abc^	4.99^ab^	6.98	0.54
	Cp	Clay	8.3	0.173	21.3	7.8^c^	4.48^a^	0.52	0.33	19.33^a^	283.3^ab^	15555.66	54	351.3	4.2	1.17^ab^	5.25^ab^	7.08	0.5
	Fb	Clay	8.3	0.17^ab^	27.66	9.53^c^	3.78^a^	0.52	0.27	20.66^ab^	235.7^a^	15022.66	60.3	272	3.4	1.37^ab^	4.99^a^	6.98	0.5
SC system	Bs	Clay	8.20	0.16^bc^	23.50	14.2^ab^	3.05^bc^	0.52	0.65	12.33^b^	271.3^ab^	15142.3	60.00	365	3.46	0.49^c^	3.42^bc^	8.75	0.55
	Ba	Clay	8.30	0.16^bc^	28.83	15.23^a^	2.75^c^	0.57	0.52	16.66^b^	245^ab^	14326.6	55.33	321.33	3.23	0.41^c^	2.61^c^	8.05	0.62
	Dw	Clay	8.23	0.15^c^	26.93	14.86^ab^	2.71^c^	0.51	0.62	12.66^b^	246^ab^	13296.3	64.67	344.33	3.87	0.41^c^	2.47^c^	8.34	0.31
	Cp	Clay	8.3	0.15^c^	28.83	15.23^a^	2.7^c^	0.57	0.52	16.66 ^b^	245^b^	14326.66	55.3	321.3	3.2	0.40^c^	2.45^c^	8.05	0.6
	Fb	Clay	8.2	0.16^bc^	26.93	14.86^ab^	2.71^bc^	0.5	0.62	12.66 ^b^	246^ab^	13296.33	64.7	344.3	3.9	0.42^c^	2.63^c^	8.34	0.3

Values labeled with the same superscript letters are not significantly different (*P* ≤ 0.05). Bs, bulk soil; Ba, barely; Dw, durum wheat; Cp, chickpeas; Fb, faba been.

### DNA extraction, library preparation, and Illumina sequencing

Total DNA was extracted from rhizosphere and bulk soil samples using MO BIO PowerSoil DNA extraction kit and quantified using NanoDrop System (2000; Thermo Scientific). For MiSeq Illumina sequencing (paired-end 2 × 150 bp), DNA was shipped to the platform of Molecular Research Laboratory (Texas, United States). The mixtures of bacterial and archaeal 16S rRNA gene amplicons (bTEFAP®) were generated using a universal primer set (515F/806R), as previously described by Dowd et al. (2008), while those of the ITS region (ITS1F/ITS2R) of fungal ribosomal DNA were achieved following the approach Gargouri et al. (2021). All sequenced reads were deposited in the NCBI Sequence Read Archive (SRA) under the BioProject numbers PRJNA748872 and PRJNA749161 for prokaryotes and fungi, respectively.

### Microbial community analysis

To maximize the quantity and quality of reliable sequences, we used the custom scripts described by Santos-Medellín (Santos-Medellín et al., 2017). First, we demultiplexed the paired-end reads, and then we removed primer regions with Cutadapt (v1.14; Martin, 2011) following specific conditions such as: minimum overlap of 10, a minimum final sequence length of 10, and a quality trim score of 10. Afterwards, we assembled reads into single sequences with PANDAseq (Masella et al., 2012). We removed the flanking ribosomal small subunit and 5.8S regions with ITSx (Bengtsson-Palme et al., 2013) and detected chimeric sequences with usearch61 (Edgar, 2010). OTU clustering at 97% identity was performed with the QIIME (Caporaso et al., 2010) implementation of UCLUST (version 10.0) using an open reference strategy against version 7 of the UNITE database^1^ (Kõljalg et al., 2013) and allowing reverse strand matching. A 0.65 similarity threshold was set for the taxonomy assignment. Before analysis, we discarded all OTUs (Blaxter et al., 2005) classified as *Plantae* or *Protista* from the OTU table. Final OTUs were taxonomically classified using BLASTn by means of the BLAST algorithm^2^ and compiled into each taxonomic level into both “counts” and “percentage” files. Count files contain the actual number of sequences, while the percent files contain the relative percentage of sequences within each sample that map to the designated taxonomic classification. Example, if there are 1,000 sequences and 100 of the sequences are classified as *Fusarium* then we represent this as *Fusarium* being 10% of that sample. Prior to any analyses, samples were rarefied to the lowest number of reads for all samples with a threshold of >10,000 reads, using Quantitative Insights QIIME’s function single_rarefaction.py, and samples with less than 10,000 reads were discarded and not included in downstream analyses. A subsequent analysis of diversity was performed based on the output-normalized data. QIIME software (version 1.9.1) was used to compute the alpha diversity indices of Shannon, and the Observed OTU based on OTU numbers to study the diversity and structure of the microbial community of each sample. The Shannon-Weiner index value calculated for different crops not only represent the species richness but also the species evenness in each agrosystem. In order to evaluate the effect of the farming systems on soil microbial community structure, the beta diversity was analyzed using one-way analysis of Similarities (ANOSIM) based on Bray-Curtis matrix via 999 permutations and further illustrated by principal coordinate analysis (PCoA) based on Euclidean distance-matrix using the *cmdscale* function in R (Langfelder and Horvath, 2012). The ANOSIM test statistic (R) ranges from 0 to 1, where the value 0 indicates random grouping between samples and the value 1 indicates 100% dissimilarity between samples (Clarke, 1993). Unique and shared operational taxonomic units (OTUs) among the two farming systems were defined by a Venn-diagram analysis constructed using the Venn Diagram package in R version (1.12.0). All heat maps were drawn by the *aheatmap* function in the NMF package in R version (0.22.0). The linear discriminant analysis (LDA) effect size (LEfSe) algorithm method was applied to the OTUs table at http://huttenhower.sph.harvard.edu/galaxy/ to identify the discriminant prokaryotic or fungal clade at the family level between study groups on Cumulative Sum Scaling’s normalized data (Segata et al., 2011). Predicted functions based on detected prokaryotic taxa were determined using Functional Annotation of Prokaryotic Taxa v.1.0 (FAPROTAX; Louca et al., 2016).

### Co-occurrence network analysis of the core microbiome and identification of microbial hubs

A co-occurrence network analysis was performed for each core microbiome associated with crop rhizosphere to explore the significant relations among the OTUs (Weiss et al., 2016), using CoNet in Cytoscape 3.8.2 (Faust and Raes, 2016) and visualized via Gephi 0.9.2 (Bastian et al., 2009). We filtered out the OTUs with frequencies less than 0.05, then combined an ensemble of the Pearson and Spearman correlation coefficients and the Bray–Curtis and Kullback–Leibler dissimilarity indices to build the network. We computed the edge-specific permutation and bootstrap score distributions with 1,000 iterations to determine the statistical significance of the co-occurrence and mutual exclusions. The nodes of each network are colored based on phylum or class affiliation (99%) and sized based on the degree of connection. Specific geometric shapes have been chosen for each microbiome species type. All *p-*values were adjusted for multiple testing using the Benjamini and Hochberg FDR controlling procedure (Benjamini et al., 2006). Statistical differences between the degrees of connection in the co-occurrence networks for crops rhizosphere nodes were analyzed. To visualize the taxonomy of the most connected nodes, we selected the nodes with a connection frequency of more than 75%. The most prevalent nodes identified based on the combined score of high mean degree, high closeness centrality, and low betweenness centrality were identified as “hubs” or “keystone.” Otherwise, they are named connectors.

### Statistical analysis

Statistical analyses were performed using the SPSS statistics 25 analytical software (IBM Corp. 2017). Mean comparison procedures of crop growth and the physicochemical parameters of soil between the two types of agrosystems (AF, SC) were carried out with one-way ANOVA using the Tukey HSD *post-hoc* test (*p* < 0.05). A mixed-model ANOVA with two fixed factors (species and system) was performed on dry matter (DM) plant biomass. The relative importance of the soil parameters dissimilarities and soil microbial taxonomic compositions (OTU level) contributing to the variation in the dry matter production was further identified by redundancy analysis (RDA) and a subsequent Monte Carlo test (999 permutations). It was performed using *rda* and *permutest* function of the vegan package in R version (2.0-2). The goodness-of-fit (R^2^) and associated statistical significance (value of *p*) of each SOM compound were verified using the *envfit* function in vegan (Oksanen et al., 2020).

## Results

### Physico-chemical characteristics of soils and dry matter production

**Figure 2:**
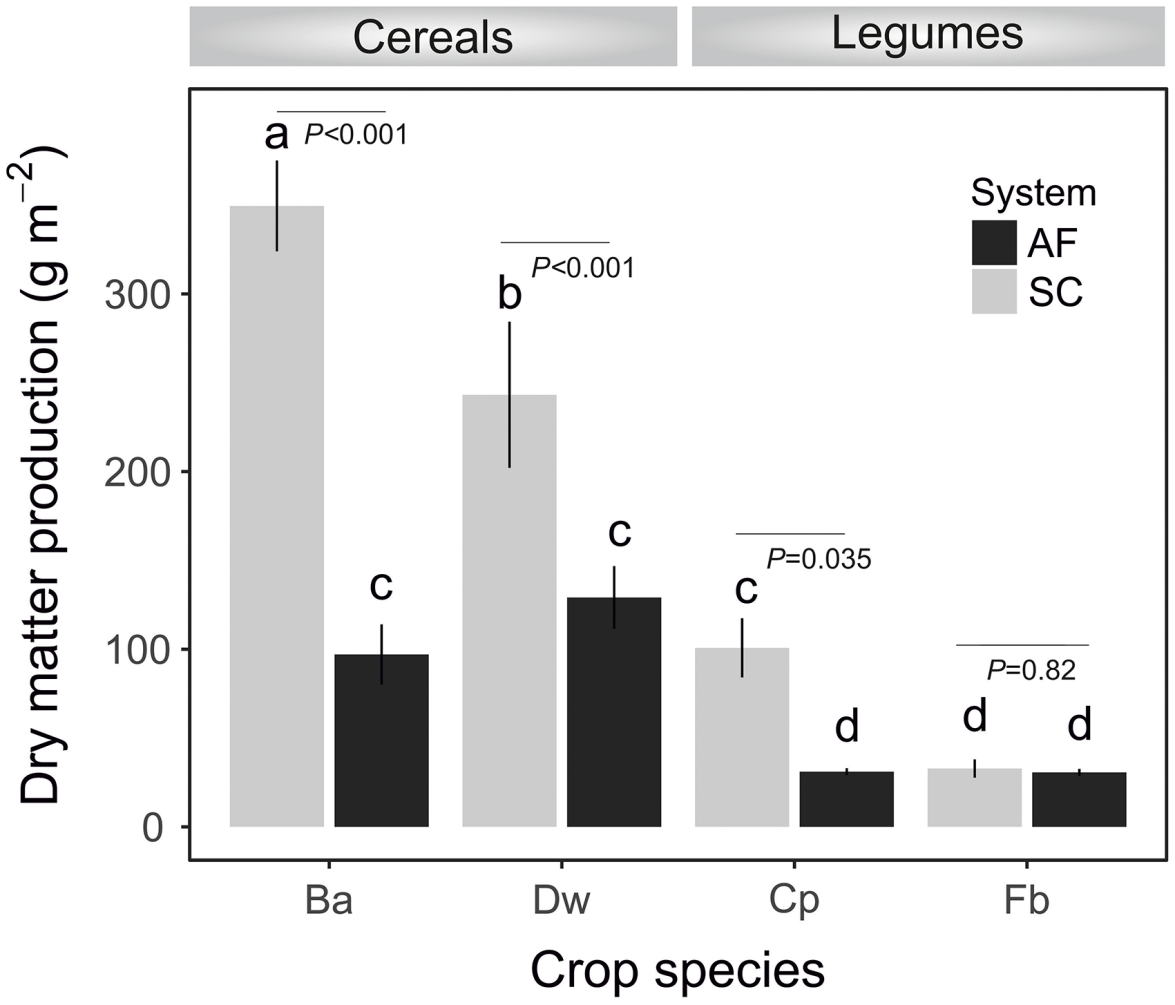
Dry matter production at the flowering stage of crops (Ba, barley; Dw, durum wheat; Cp, chickpea; Fb, Faba bean) in agroforestry system (AF) and full sun cropping (SC) systems. Bars indicate the mean biomass *± SE*. Letters show significant differences from the ANOVA and *post-hoc* Tukey test.

Soil characteristics differed between SC and AF systems. Both agrosystems had a similar pH (8.2) and clay texture. The total organic content (TOC) percentage was two times higher, and the percentage of active limestone was two times lower in AF than in SC systems (Table 1). A significant difference in dry matter production (DM) was recorded between and within cereal and legumes species (Figure 2). Species identity, the type of agrosystem, and their interaction have a significant effect (*p* < 0.0001) on DM (Supplementary Table S1). DM of barley (*p* < 0.0001), wheat (*p* = 0.0179), and chickpea (*p* = 0.0142) were lower in AF than in SC systems. Nevertheless, the DM of faba bean was similar in both systems (Figure 2; Supplementary Table S1).

### Prokaryotic and fungal taxonomic richness and diversity

**Figure 3:**
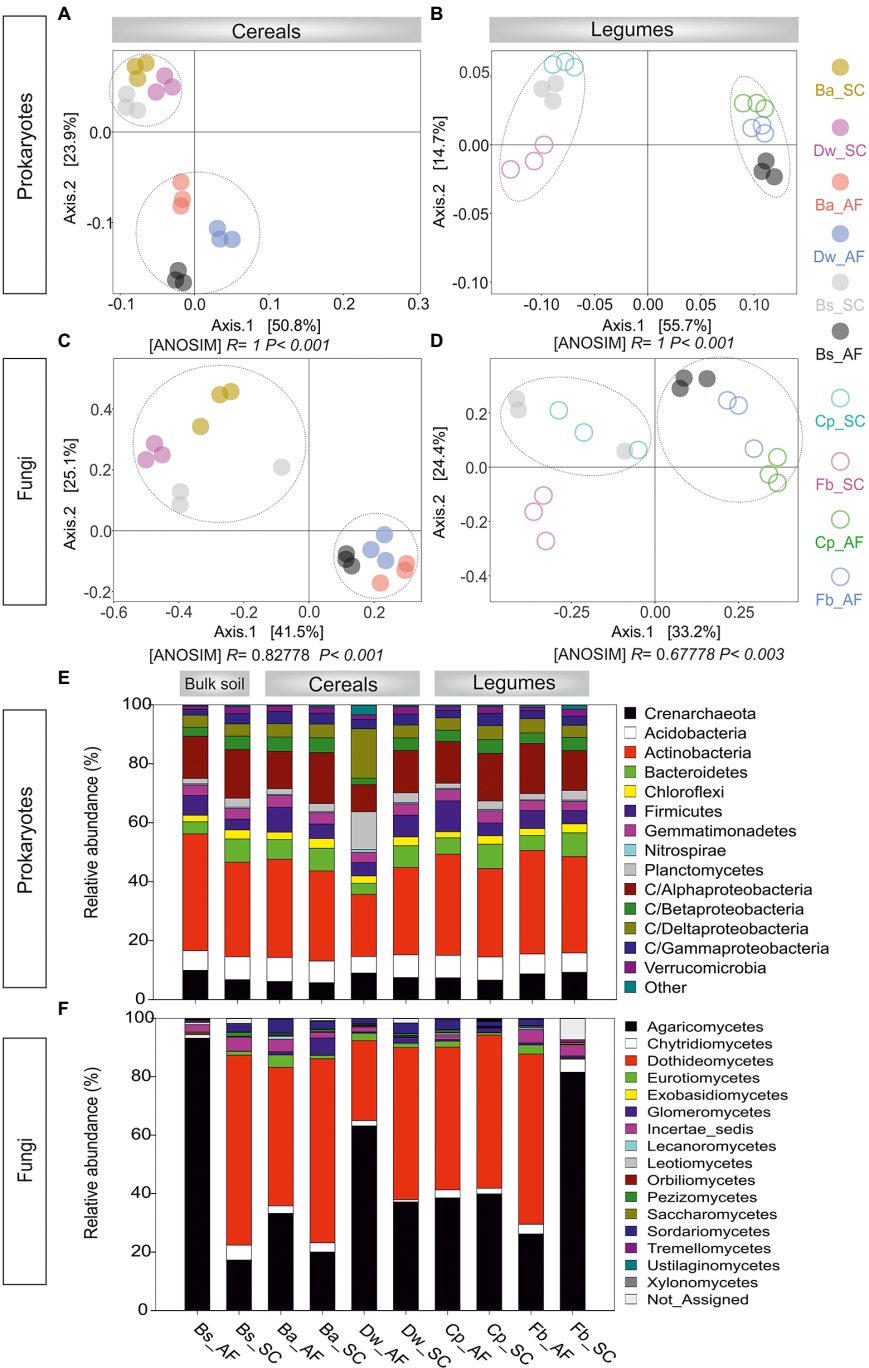
Principal coordinates analysis (PCoA) plot for the **(A)** procaryotic and **(B)** fungal communities associated with the rhizosphere of cereals and legumes. **(C)** Relative abundance of procaryotic and **(D)** fungal rhizosphere communities across the different crops. AF, agroforestry system; SC, full sun cropping; Bs, bulk soil; Ba, barley; Dw, durum wheat; Cp, chickpea; Fb, Faba bean. The relative abundance of each phylum, class, and order in different rhizosphere samples was summarized in histogram graphs using R software environment (v.3.2.5) and the significant differences were determined using Tukey’s HSD statistical test.

Through high-throughput sequencing analyses of 16S rDNA and ITS rDNA, 2,781.578 prokaryotic sequences and 1,421.271 fungal sequences were read and generated 2,592 prokaryotic OTUs and 571 fungal OTUs. The rarefaction curves of both prokaryotes and fungal communities approached an asymptote, indicating the sufficiency of our sequencing depths (Supplementary Figure S3). For the prokaryotes, species richness and Shannon diversity index were both significantly lower in AF than in SC (*p* < 0.05), whether for bulk soil or the rhizosphere of cereals and legumes crops (Supplementary Figure S4A). For the fungal community, the bulk soil microbiome was equally diverse in AF and SC, while the alpha diversity of crops rhizosphere microbiome (RM) was significantly lower in AF than in the SC system (Supplementary Figure S4B). PCoA analyses on Bray–Curtis dissimilarity matrixes corroborated these results. Plots showed that the microbial communities were clustered only in an agrosystem-dependent manner along the PC1 axis (AF vs SC; *p* < 0.01, ANOSIM; Figure 3). Moreover, the fungal legumes plot (Fb_SC) formed a significantly distinct community cluster (Figure 3D), suggesting that legumes’ fungal microbiome is species-specific.

### Prokaryotic and fungal taxonomic profiles abundance, dominant taxa, specific and shared microbial assemblages between sun cropping and agroforestry systems

In prokaryotic profiling (Figure 3E; Supplementary Table S5), we detected 61 classes belonging to 20 phyla. Soil RM structure were mainly represented by *Actinobacteria* (21.0%–35.0%), *Proteobacteria* (23.4%–31.3%), *Crenarchaeota* (5.7%–9.2%), *Acidobacteria* (5.6%–8.1%), *Bacteroidetes* (3.7–8.2), *Planctomycetes* (1.6%–12.8%), *Firmicutes* (4.3%–10.4%), *Chloroflexi* (2.2%–3.2%), and *Verrucomicrobia* (1.0%–2.5%). Microbial phylogenetic profiling showed that *Acidobacteria*, *Chloroflexi*, *Gemmatimonadetes*, and *Betaproteobacteria* were less abundant in AF than in SC. Furthermore, each cereal or legume species displayed a species-specific response to AF with significant changes in different phylum and/or class compositions. For barley, *Gammaproteobacteria* was significantly enriched in AF than in the SC system, whereas *Verrucomicrobia* and *Bacteroidetes* were significantly depleted. For durum wheat, a relative abundance of *Planctomycetes* and *Deltaproteobacteria* was enriched by 70% in AF *vs* SC, while *Firmicutes* and *Actinobacteria* were decreased. Within legumes RM, chickpea was enriched by 58% of *Firmicutes* in AF than in SC. For faba bean, *Alphaproteobacteria* was enriched in AF than in SC of faba bean, while *Crenarchaeota* was depleted.

In fungal profiling (Figure 3F; Supplementary Table S6), we detected 16 fungal classes belonging to 5 phyla. The *Agaricomycetes* class was the most abundant (20.0%–81.5%) followed by *Dothideomycetes* (0.1%–62.9%), *Glomeromycetes* (0.4%–5.9%), *Sordariomycetes* (0.1%–4.7%), *Chytridiomycetes* (1.7%–4.4%), *Eurotiomycetes* (0.1%–4.2%), and *Lecanoromycetes* (0.1%–1.0%). For barley, *Lecanoromycetes* and *Sordariomycetes* were 2 to 4 times more abundant in AF than in SC. *Chytridiomycetes* were two times more abundant in AF than in SC for durum wheat. For both legumes, *Sordariomycetes* were more abundant in AF than in SC. However, for faba bean, *Dothideomycetes* were enriched in AF of faba bean while *Agaricomycetes* were depleted. In addition, while *Glomeromycetes,* the only arbuscular mycorrhizal fungi class, were generally less abundant in AF than in SC, no significant changes were observed between AF and SC for faba bean (*p* < 0.05).

Venn diagram showing the number of shared and unique OTUs for the (a) prokaryotic communities (Supplementary Figure S5A) and (b) fungal communities (Supplementary Figure S5B). For the shared OTUs, 95% of identified prokaryotic OTUs were shared across each crop and its associated bulk soil, while only 50% of fungal OTUs were shared. For the unique OTUs, the number of fungal OTUs was considerably higher than the number of bacterial OTUs. Besides, unique fungal OTUs number was lower in AF than in SC of cereal or legume species.

**Figure 4:**
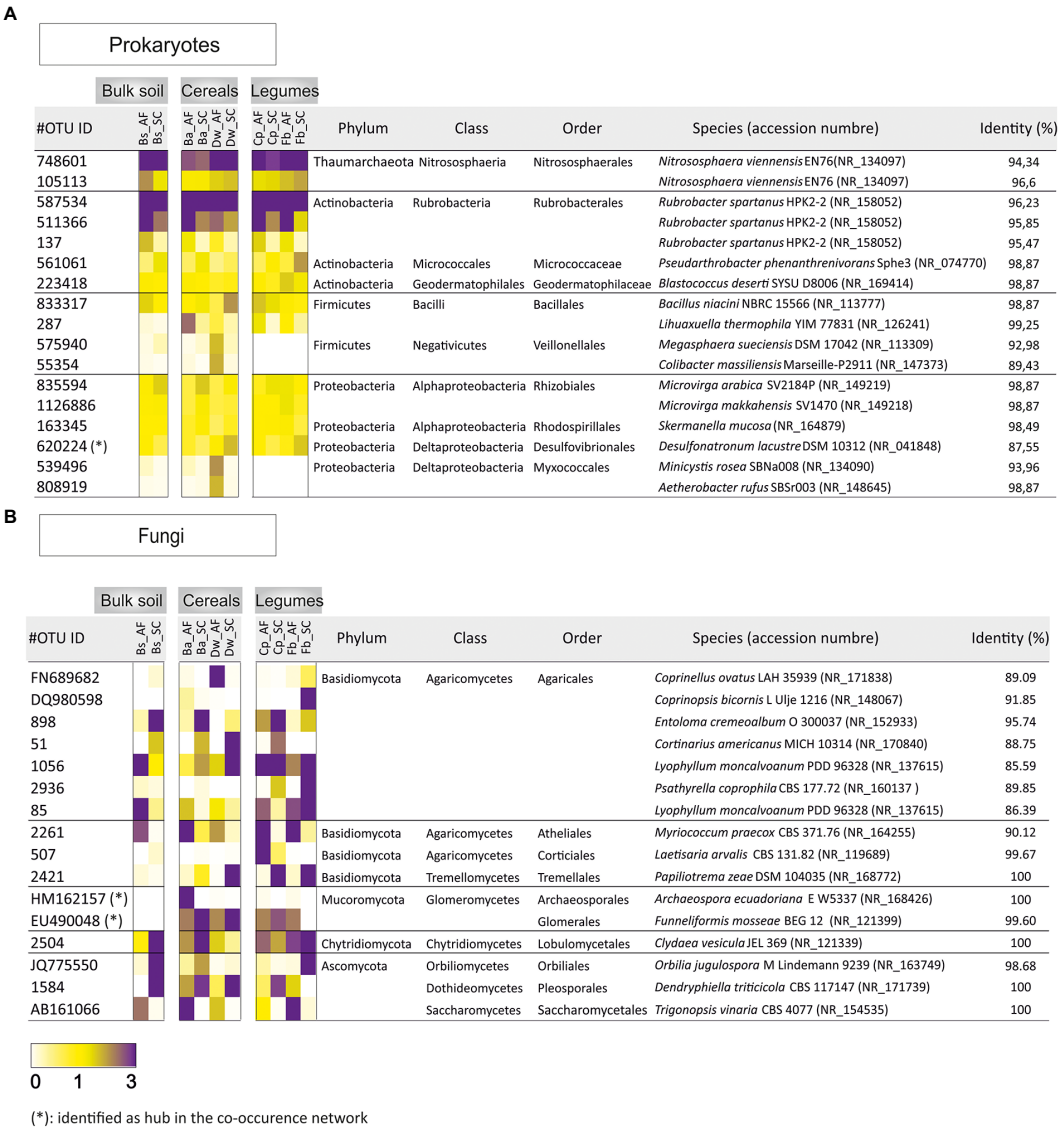
Heat map exhibiting the relative abundance of dominant **(A)** prokaryotic (>1% of all sequences) and **(B)** fungal (>3% of all sequences) in agroforestry system (AF) and full sun cropping (SC) samples. Bs, bulk soil; Ba, barley; Dw, durum wheat; Cp, chickpea; Fb, Faba bean.

The 17 and 16 most abundant prokaryotic and fungal OTUs were plotted as heat map diagrams to identify the dominant taxa between the two types of agrosystems and within the different crop species (Figure 4). At the order level, prokaryotic OTUs (>1%) were abundant in AC *vs* SC: *Bacillales* (OTU287) were detected in soil rhizosphere of barley and both legumes’ species. OTUs related to *Veillonellales* (OTU575940 and OTU55354) and *Myxococcales* (OTU539496 and OTU808919) were increased in the soil rhizosphere of durum wheat. *Micrococcaceae* (OTU561061) was abundant only in the soil rhizosphere of the faba bean (Figure 4A). For fungal OTUs (>3%): Glomerales (EU490048) were abundant in barley, chickpea, and faba bean soil rhizospheres. *Lobulomycetales* (OTU2504) were increased in durum wheat and chickpea soil rhizosphere. *Agaricales* (OTUFN689682), *Corticiales* (OTU507), and *Pleosporales* (OTU1584) were abundant in durum wheat, chickpea, and faba bean, respectively (Figure 4B).

LEfSe analysis (LDA > 3) results revealed that bulk soils and crops species elicited unique sets of bacterial/fungal biomarkers between the two types of agrosystems (Supplementary Figures S6A,B).

### Functional prediction analysis

FAPROTAX analysis showed that 14.5% (369 out of 2,533) of all bacterial OTUs were assigned to at least one ecological type (Supplementary Figure S7). A total of 32 metabolic and ecological types were predicted. Community function varied across cereals and legumes varieties. Barley samples presented more than two-fold of sequences abundance associated with hydrocarbon degradation. Durum wheat samples showed an increase of sequences related to aerobic nitrite oxidation, chloroplasts, xylanolysis, cellulolysis in AF than SC. Faba bean samples increased 11 functional groups mainly linked to the biogeochemical N-cycles (e.g., nitrogen respiration, nitrate denitrification, denitrification) and photoheterotrophy. In contrast, chickpea samples were dominated by functional groups linked to the biogeochemical cycles of carbon (hydrocarbon degradation, aromatic hydrocarbon degradation, aliphatic non-methane hydrocarbon degradation, photoautotrophy, phototrophy).

### Relationship between dry matter production, soil properties, and rhizosphere microbial community

**Figure 5:**
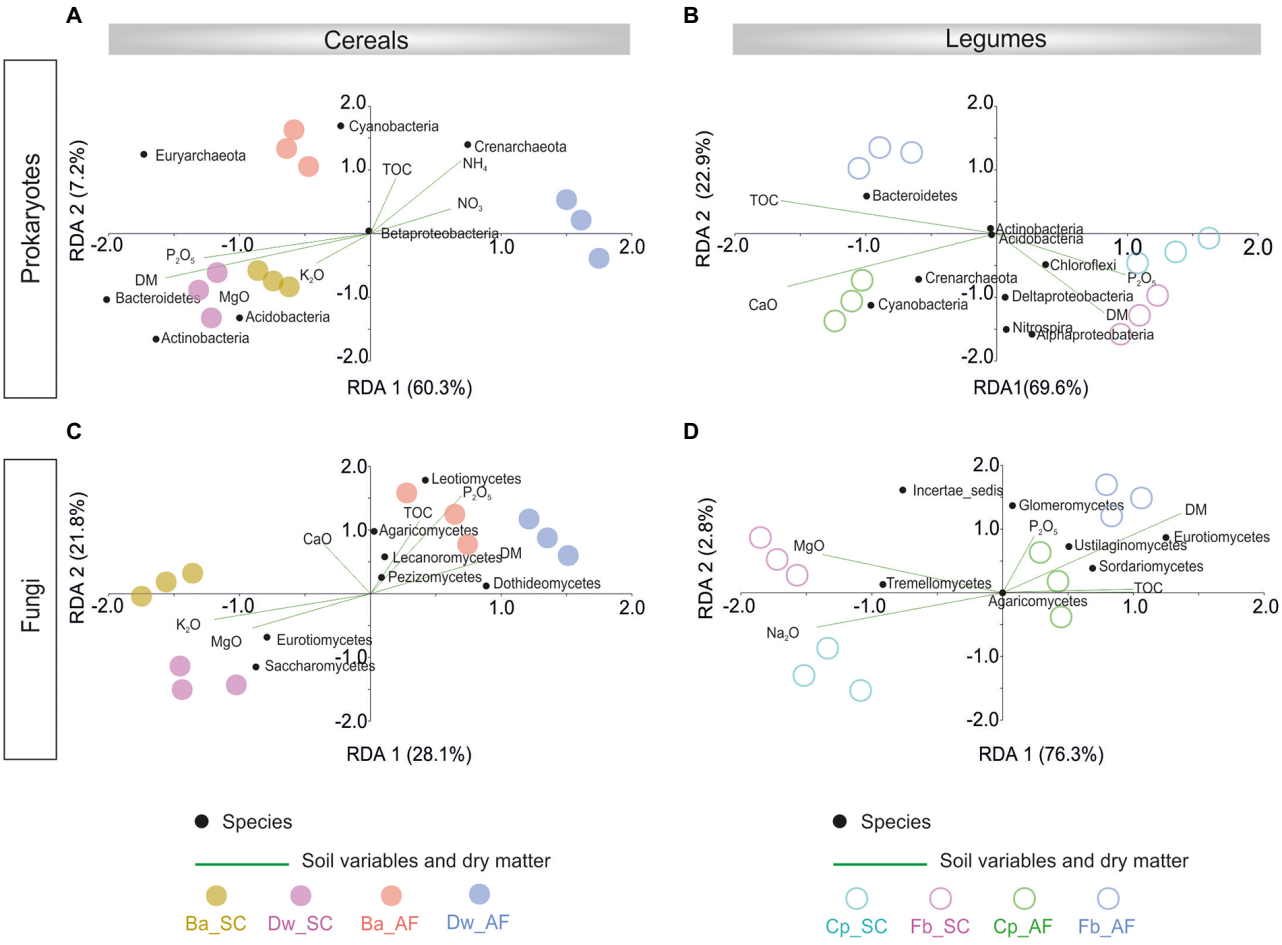
Redundancy analysis (RDA) showing variation in abundant rhizosphere prokaryotes phylum/class in cereals **(A)** and legumes **(B)** crops and abundant rhizosphere fungi in cereals **(C)** and legume **(D)** explained by crops dry matter (DM) production and soil physicochemical parameters. Significant influencing factors derived from Nonparametric Spearman correlations are shown (*p* < 0.05). AF, agroforestry system; SC, full sun cropping; Ba, barley; Dw, durum wheat; Cp, chickpea; Fb, Faba bean; TOC, total organic carbon.

The redundancy analysis (RDA) showed that the prokaryotic community was correlated with dry matter plant biomass (DM) in the SC system, and the fungal community was associated with DM in the AF system (Figure 5; Supplementary Table S2). The microbial community-DM correlation was specific to each crop species. In addition, %TOC was correlated only with the fungal community from AF samples.

### Meta-network topology and core microbiome interactions

Each core microbiome correlated prokaryotes OTUs (bacteria + archaea), fungal OTUs (fungi + AMF), and soil parameters. A significantly higher number of mutual exclusions interactions than co-occurrence has been recorded in all samples (Supplementary Table S3).

**Figure 6:**
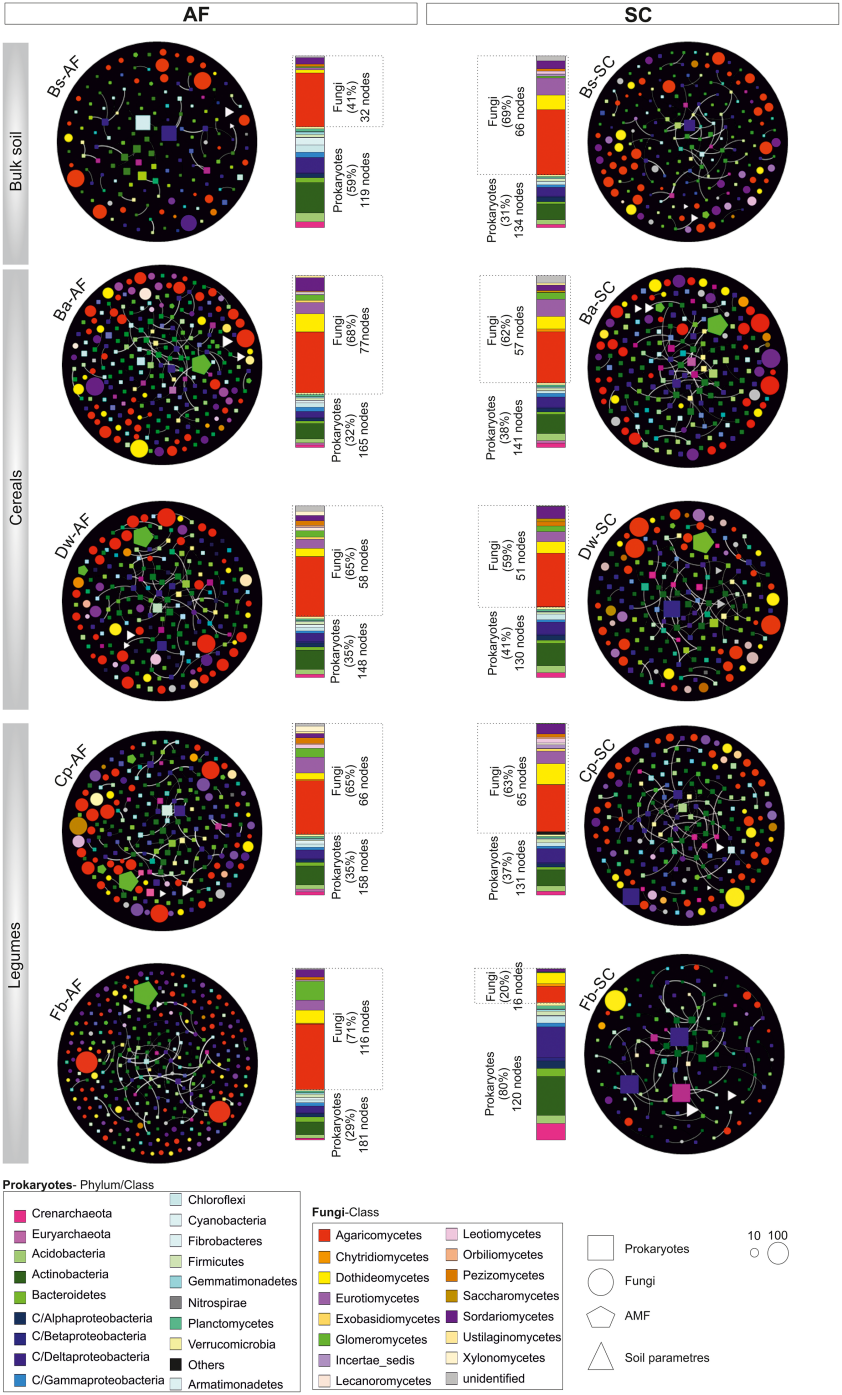
Network topology plots of the core microbial communities associated with root systems of cereals and legumes crops in an agroforestry system (AF) and full sun cropping (SC) samples. The nodes of each network are colored based on phylum or class affiliation (99%) and sized based on the degree of connection. For each microbiome species type, specific geometric shapes have been chosen (square, procaryotes; circle, fungi; AMF, pentagon), and the triangle shape has been selected for physiochemical soil parameters. White lines represent the thickness of the edge connecting the nodes. Relative abundance of those nodes with degree of connection is reported at the phylum/class level in the bar chart for each sample. Bs, bulk soil; Ba, barley; Dw, durum wheat; Cp, chickpea; Fb, Faba bean.

The bulk soil network has fewer nodes and edges in AF *vs* SC (Figure 6), whereas, in all crop networks, the number of nodes and their interconnections were higher in AF samples and had a higher number of communities as compared to SC (Supplementary Table S4). Interestingly, we noticed a significant difference between cereals and legumes meta-network topology following the type of agrosystem (Supplementary Table S4). The average path lengths were higher in AF than in SC within cereals networks. Furthermore, a significant decrease of betweenness centralization was observed, indicating the peripheral node’s location and the unstructured distribution of these networks. Conversely, legumes networks in the AF system demonstrate a lower average path length and a higher betweenness centralization than SC networks *p* < 0.05 (Supplementary Table S7), revealing a cohesive and high compactness relationship among AF-legumes communities. Remarkably, faba bean in AF demonstrates a high network heterogeneity (2.78) compared to all other networks. Altogether, these results highlighted that the legume network has a more connected community than the cereal network in the AF system, except the faba bean network.

### Prokaryotic and fungal hubs of each crop network

The core microbiome of both types of agrosystem networks was composed mainly of prokaryotic hubs (connectors), while the highly connected hubs (keystones) of these networks were exclusively fungal taxa (excepting those of faba bean in SC; Figure 6). The two types of agrosystems shift the network structure of rhizosphere microbiota and uncover that each type of agrosystem harbors distinct hubs (Table 2). Thirteen cereals-associated taxa and 11 legumes-associated taxa were identified as keystones within the two types of agrosystems. Interestingly, we observed that the hubs involved in SC were distinct from those identified in AF. All networks are dominated by fungal keystones belonging to the *Ascomycota*, *Basidiomycota*, and *Mucoromycota* phyla. Conversely, the faba bean network in SC was dominated by prokaryotic keystone species (*Desulfonatronum*, *Desulfomonile*, and *Nitrososphaera*), while the faba bean network in AF was dominated by *Entoloma* and *Bovista* genera. Moreover, most of the hubs taxa were relatively low abundant, except two AMF genera: *Funneliformis* dominated in the barley (SC and AF), chickpea (AF) and faba bean (AF) networks, and *Archaeospora* in the durum wheat (AF and SC) networks (Table 2).

**Table 2:** Top identified keystone taxa for specific connected operational taxonomic unit (OTU) in the rhizosphere of cereals and legumes crops under agroforestry system (AF) and sun cropping (SC) systems.

OTU	Degree of connectivity										Taxonomy (phylum/class)	Species (accession number)		Similarity (%)
	Bulk soil		Cereals				Legumes						
	Bs_AF	Bs_SC	Ba_AF	Ba_SC	Dw_AF	Dw_SC	Cp_AF	Cp_SC	Fb_AF	Fb_SC			
620224 (*)						20				20	Deltaproteobacteria	*Desulfonatronum lacustre* DSM 10312 (NR_041848)	87
JQ666641				20							Agaricomycetes	*Cortinarius calyptratus* MICH 10328 (NR_130201)	79
AB103380				20							Sordariomycetes	*Purpureocillium lilacinum* NRRL 895 (NR_165946)	100
2196				20		20					Agaricomycetes	*Inosperma neobrunnescens var. leucothelotum* WTU ACAD11597 (NR_153187)	98
EU490048 (*)			32	20			31		135		Glomeromycetes	*Funneliformis mosseae* BEG 12 (NR_121399)	97
GU056002			32								Sordariomycetes	*Trichoderma crassum* DAOM 164916 (NR_134370)	98
4853			32								Dothideomycetes	*Neocamarosporium chersinae* (NR_154261)	94
4277			32				31				Agaricomycetes	*Tomentella agbassaensis* M SYN 981 (NR_119638)	91
819						20					Agaricomycetes	*Psathyrella complutensis* AH 33713 (NR_158909)	92
4867						20					Agaricomycetes	*Tricholoma boreosulphurescens* (NR_159050)	88
HM162157 (*)					22	20					Glomeromycetes	*Archaeospora ecuadoriana* (E W5337 NR_168426)	100
HM240195					22						Agaricomycetes	*Inocybe viscata* PDD 27109 (NR_119772)	100
FJ581422					22						Agaricomycetes	*Tomentella agbassaensis* M SYN 981 (NR_119638)	89
FJ553314										20	Dothideomycetes	*Neocucurbitaria rhamnioides* (NR_156361)	90
4051670								24		20	Deltaproteobacteria	*Desulfomonile tiedjei* DSM 6799 (NR_074118)	89
4400135										20	Nitrososphaeria	*Nitrososphaera viennensis* (EN76 NR_134097)	95
1433									135		Agaricomycetes	*Entoloma cremeoalbum* O 300037 (NR_152933)	83
EU490090									135		Agaricomycetes	*Bovista psammophila* UFRN Fungos (NR_166304)	87
AM901853								24			Dothideomycetes	*Neosetophoma rosarum* (NR_157524)	99
346							31				Agaricomycetes	*Gymnopilus speciosissimus* (NR_169697)	90
2274							31				Saccharomycetes	*Kodamaea anthophila* CBS 8494 (NR_155239)	98

Bs, bulk soil; Ba, barley; Dw, durum wheat; Cp, chickpeas; Fb, Faba beans.

* OTU > 1% (Prokaryotes) and OTU > 3% (Fungi).

## Discussion

Contrary to previous studies focused on the effect of cover crops on the olive (RM; Moreno et al., 2009; Sofo et al., 2010, 2014; Montes-Borrego et al., 2014), our field-based comparative study reveals for the first time if olive trees affect in turn the microbial profiles of allied cash/annual crops (i.e., cereals or legumes species), under organic management, and low rainfall conditions. Interestingly, our results demonstrated that bulk soil had lower prokaryotic diversity in (AF) than (SC), which is in line with studies reporting that fields with trees had significantly lower bacterial diversity compared with open-field without trees (Banerjee et al., 2016). This alpha-diversity between AF and SC did not differ significantly on richness and evenness. However, relatively no differences in prokaryotic diversity was observed with the different species in AF and SC, which is in line with a study showing that species identity does not necessarily promote bacterial alpha diversity (Prober et al., 2015).

In contrast, fungal diversity in bulk soil was not affected by the presence of olive trees. This result is also consistent with the recent investigation of Rivest et al. (2019), showing that soil fungi from the bulk soil was weakly related to the tree presence and properties. Inversely, a significant difference in fungal diversity has been generated between the two agrosystems (AF vs. SC), with a similar response for all crop species. Thus, our findings determined that annual crops were the primary driver for soil fungi diversity in both types of agrosystems and that it is likely high redundancy in fungi that can optimally use these ecological niches, suggesting that the fungal communities were possibly more influenced by the type of agrosystem than the prokaryotic one.

Moreover, beta diversity analysis also showed a dissimilarity of microbial communities between AF and SC, with the unique exception of the fungal communities associated with faba bean in SC, supporting the general rule that soil microbes responded significantly to the tree presence (Rivest et al., 2019). However, it is essential to emphasize that trees were not only drivers of soil microbiological diversity, suggesting that soil properties or extreme climatic conditions were also involved in structuring soil microbial assemblages (Beugnon et al., 2021). Notably, based on redundancy analysis, our data showed an increase of the total organic carbon (TOC) correlated with the diversity of microbial communities in AF samples. Here, the rise in TOC was undoubtedly one of the responsible factors for the observed differences in microbial beta diversity between AF and SC systems. Recently, Abbasi Surki et al. (2020b) reported that the AF systems sequestration of carbon in the soil and offer higher soil moisture to the next crop. Thus, we can suggest that the soil microbial communities of AF can be structured by effects of the above-ground olive leaf litterfall, woody debris and/ or olive root turn-over, which is not the case in SC. Here, one of explanations can be drawn that olive trees indirectly affect the soil rhizosphere beta diversity of microbial communities associated with annual crops via the increased carbon. Our results are in agreement with previous studies reporting relatively low levels of bacterial and fungal evenness in soils with high carbon content compared to soils with low carbon content (Delgado-Baquerizo and Eldridge, 2019; Větrovský et al., 2019; Bastida et al., 2021).

Furthermore, our results support the notion that, as soil C content increases, the abundance of dominant taxa are promoted, which in turn reduces the diversity of subordinate taxa *via* competitive exclusion, resulting in an overall reduction in species evenness in AF as compared to SC (Bastida et al., 2021; Saghaï et al., 2022). In addition, this reduction of soil microbial diversity associated with annual plants rhizosphere was concomitant to a decrease of plant biomass at the flowering stage, except the unchanging biomass of faba bean plants between AF and SC. Because of that, we do not believe that this biomass reduction was only due to the effect of shade provided by olive trees, as was reported previously (Inurreta-Aguirre et al., 2018; Temani et al., 2021). There is a link between the diversity of RM and the biomass production of plants that together are responsible for enhancing soil carbon assimilation, which is in line with several previous investigations (De Deyn et al., 2011; Merino et al., 2015).

Shared prokaryotes OTUs (95%) between AF and SC were not affected by the presence of an olive tree or crop species. At least two hypotheses can explain this: (i) the soil rhizosphere is the primary driver of bacterial and archaeal communities, which is supported by previous works reporting that soil type strongly influences bacterial and archaeal communities irrespective of the absence or presence of vegetation (Lupatini et al., 2013; Suleiman et al., 2017) or (ii) there is a well-adapted soil prokaryote community, independent of vegetation cover or soil properties (e.g., high % TOC in AF plots) that can exhibit an outstanding level of similarity despite several modifications in soil properties (Marshall et al., 2011; Wallenius et al., 2011). In contrast, fungal communities consisted of a lower proportion of shared OTUs (an average of 50%) between the two types of agrosystems and a smaller number of specific OTUs per soil crop species among AF samples than SC samples, suggesting the importance and the impact of the type of agrosystem (AF or SC) specifically on the fungal richness.

Our study determined that the microbiome abundance profile associated with annual plants rhizosphere is chiefly influenced by the type of agrosystem where prokaryote and fungi profiles in AF are distinct from those in SC. Host species have a low level of selection within the two types of agrosystems, with a certain degree of host-specificity (i.e., each crop species of cereals or legumes harbored specific microbial taxa with distinct relative abundance between AF and SC groups). All this evidence suggests that the microbes inhabiting the soil rhizosphere were shaped by both the host (crop species) and biotic interactions (presence of olive trees or not). It is reasonable as the soil rhizosphere was supposed to be the interface of interactions (Kumar and Dubey, 2020). Indeed, shared OTUs belonging to *Actinobacteria, Proteobacteria*, and *Firmicutes* as bacterial phyla and *Basidiomycota*, *Ascomycota*, *Mucoromycota*, and *Chytridiomycota* as fungal phyla were highly enriched in AF than SC (Figure 4). These phyla were reported to be the most abundant phyla defining the rhizosphere of olive-associated microbial communities (Fausto et al., 2019; Fernández-González et al., 2019, 2020), suggesting that the microbial communities associated with cereals and legumes in the AF system were probably recruited from the surrounding olive orchard microbial communities. This suggestion confirms one of the central hypotheses of this study that olive-based AF plays a crucial role in determining the RM profile of annual crops.

The network analysis revealed that the connectivity in AF networks was higher than in the SC networks. Thus, it might be linked to the remarkable resilience capabilities of the AF RM to stress disturbances or invasion of exogenous microbes (Mendes et al., 2018; Banerjee et al., 2019; Liao et al., 2019). It has been previously shown that complex networks with more excellent connectivity are more robust to environmental perturbations than simple networks with lower connectivity (Santolini and Barabási, 2018). Increased network complexity may result from enhanced resource availability, such as elevated CO_2_ (Zhou et al., 2011) and soil fertility (Guo et al., 2020). Interestingly, higher connectivity in legumes AF networks was observed (Supplementary Table S4). Because of their N_2_-fixation capacity, legumes positively influence soil fertility and are less competitive for soil resources than cereals, and their RM may have outcompeted indigenous strains by constituting a cohesive network under AF conditions (Siczek et al., 2018; Vanlauwe et al., 2019). In our comparative study, cereals produced significantly more above-ground biomass. Although this study did not report effects on soil moisture, we assumed that the higher DM production of cereals compared to legumes was associated with higher consumption of soil resources.

Both types of agrosystem networks harbored many prokaryotic connectors but were dominated by fungal keystones, except those of faba bean in SC (Figure 6). Unexpectedly, based on our RDA analysis, these fungal keystones were significantly correlated with DM within the AF system (Figure 5). In addition, and in one hand we demonstrated that DM was lower in AF than SC for barley, wheat, and chickpea (Figure 2) and in second hand fungal keystones within the AF system were more involved in ecological processes (i.e., litter decomposition and drought tolerance) than in promoting crop production (Banerjee et al., 2018).Thus, we hypothesized that the low DM could be attributed to the stress caused by a microbial competition between RM crops and olive tree for sharing soil resources within the AF system under rainfed conditions, further studies are needed to increase the generalizability of these findings. Furthermore, the significant DM differences between cereals species and legumes species in SC were not observed under AF conditions (Figure 2), suggesting that olive AF is the best compromise system to balance maintaining crop production and improving crop production soil fertility of annual crops.

In general, fungi are considered significant contributors to the biogeochemical cycles in the AF system. They are key litter decomposers responsible for producing extracellular hydrolytic and oxidizing enzymes leading to access to new substrates through hyphae (Bani et al., 2018) and act as indicators of abrupt environmental changes (Herren and McMahon, 2018). Indeed, we identified that the fungal keystones associated with the AF rhizosphere system consisted in part of some ectomycorrhizal fungi genera such as *Inocybe*, *Russula*, *Cortinarius*, and *Tomentella* (Gehring et al., 2017; Thiem et al., 2018; Frey, 2019; Nicolás et al., 2019) and free-living culturable fungi (*Trichoderma* and *Neocamarosporium*; Table 2) which were known to develop some strategies and mechanisms helping host plants to thrive harsh environmental conditions (Hosseyni Moghaddam et al., 2021; Zuo et al., 2021). These fungal keystones were promoted in our experimental conditions within the different networks since the field trials were conducted under severe winter drought episodes, during which rainfall amounted to about 50% less than expected, corresponding to one of the driest winters in 30 years.

We also identified two highly abundant AMF keystone taxa, *Archaeospora* and *Funneliformis* (Figure 4; Table 2). AMF symbiosis has improved plant resistance (Bahadur et al., 2019). *Archaeospora* genus was known to have high plasticity and adaptability to withstand drought stress by secondary responses, such as enhancing the soil structural stability and strengthening the soil water retention (Wang et al., 2021). *Funneliformis* is the most widely distributed symbiont assisting plants to overcome counteractive environmental conditions (El-Gazzar et al., 2020) and is identified as indicator species for physical soil disturbance (Peyret-Guzzon et al., 2016; Van Tuinen et al., 2020; Cheng et al., 2021). Thus, it suggests that these fungal keystones help barley, durum wheat, and chickpea plants exploit their ecological niche within the AF system by facing the water-scarce condition and the nutritional competition than promoting growth production. It could explain the high production observed in the SC system where these fungal keystones were most likely less subjected to competition with olive tree for soil resources and hence less % connectivity exclusion in their networks. This result stressed the critical role of fungal keystones, particularly AMF, in enhancing the productivity of cereals within the SC system. Furthermore, several studies have tried to use rhizosphere fungi as microbial inoculants in agriculture (Subrahmanyam et al., 2020). Therefore, exploring AMF keystones with the highest potential in olive AF will be a promising direction for producing bio-inoculants to manipulate cash crop-associated microbiomes in olive AF under climate change future scenarios in the semi-arid areas.

Conversely to barley, durum wheat, and chickpea networks of the SC system, the faba bean network in SC showed the lowest DM (Figure 2) and was dominated instead by prokaryotic keystones taxa (Figure 6), presenting mainly functional groups linked to the biogeochemical N-cycles (Supplementary Figure S7). The prokaryotic keystones taxa belonging to *Desulfonatronum* and *Desulfomonile* was sulfate-reducing bacteria known to establish a tightly synergistic relationship with the nitrogen fixation process and heat tolerance (Devereux, 2005). Together, this result suggested that the higher DM in SC system is most likely depend on the presence of fungal keystones.

## Conclusion

The outcomes of this investigation provide comprehensive and empirical evidence for the effective selection and assembly of contrasting crop microbiome between different types of agrosystems (AF and SC) under low rain-fed conditions. We showed that rhizosphere microbial (RM) diversity was significantly lower in AF than SC for cereals and legumes, and the presence of olive trees, the soil proprieties, and the crop species determine the soil microbial profiling of annual crops. The redundancy analysis showed that the prokaryotic community was correlated with biomass in the SC system, while the fungal community was correlated with biomass production in the AF system. We also demonstrated that the fungal keystones are crucial moderators of the core microbiome. They were involved in the ecological process within the AF system and promoting crop production within the SC system. We have identified potential key microbial factors that can be targeted for the sustainability of agroecosystems under drought stress.

Moreover, legumes networks were more cohesive than cereals networks, which may led us to hypothesize (i) that legumes RM possessed a greater tolerance to environmental conditions than cereals, (ii) and that legumes are less competitive for soil resources, and cereals are more suitable for biomass production near the olive tree. Follow-up studies now need to confirm these hypotheses and to determine the impact of cereals and legumes intercropping in AF on soil sustainability. Therefore, future investigating for intercropped cereals and legumes microbiome in AF will provide untapped opportunities to develop more sustainable and productive agroecosystems.

## Data availability statement

All sequenced reads from this study has been deposited to the NCBI Sequence Read Archive (SRA) under the BioProject numbers PRJNA748872 and PRJNA749161 for prokaryotes and fungi, respectively.

## Author contributions

ABZ, MG, KB, AM, and SS conceived and designed the study. KB coordinated the sampling and performed the soil physic-chemical analyses. FK and NM performed the molecular analyses. ABZ and MG performed the bioinformatic and statistical analyses. ABZ wrote the original draft of the manuscript. All authors contributed to the article and approved the submitted version.

## Funding

This research was carried out as part of the D4DECLIC Project, ARIMNet 2 Young Scientists Call 2017 (ERA-NET program), and Grant agreement no. 618127.

## Conflict of interest

The authors declare that the research was conducted in the absence of any commercial or financial relationships that could be construed as a potential conflict of interest.

## Publisher’s note

All claims expressed in this article are solely those of the authors and do not necessarily represent those of their affiliated organizations, or those of the publisher, the editors and the reviewers. Any product that may be evaluated in this article, or claim that may be made by its manufacturer, is not guaranteed or endorsed by the publisher.

## Footnotes

^1^ https://unite.ut.ee/repository.php

^2^ http://www.ncbi.nlm.nih.gov/
