# Supplementary material for: Olive agroforestry shapes rhizosphere microbiome networks associated with annual crops and impacts the biomass production under low-rainfed conditions
Source: Front Microbiol. 2022 Oct 28;13:977797. doi: 10.3389/fmicb.2022.977797 (PMC9650424; doi:10.3389/fmicb.2022.977797)
Supplement: Supplementary file 2 [file Data_Sheet_1.PDF]

Supplementary Material

Olive agroforestry shapes rhizosphere microbiome networks associated with annual crops and impacts the biomass production under low-rainfed conditions

Ameni Ben Zineb[1], Karim Barkaoui[2,3], Fatma Karray[4], Najla Mhiri[4], Sami Sayadi[5], Ahmed Mliki[1], Mahmoud Gargouri[1*]

[1]Laboratory of Plant Molecular Physiology, Centre of Biotechnology of Borj-Cedria, BP 901 Hammam-Lif 2050, Tunisia

[2] CIRAD, UMR ABSys, F-34398 Montpellier, France.

[3] ABSys, Univ Montpellier, CIHEAM-IAMM, CIRAD, INRAE, Institut Agro, Montpellier, France

[4]Laboratory of Environmental Bioprocesses, Centre of Biotechnology of Sfax, BP 1177, 3018 Sfax, Tunisia

[5] Biotechnology Program, Center for Sustainable Development, College of Arts and Sciences, Qatar University, Doha 2713, Qatar

1 Supplementary Data

Soil analysis

Soil properties were measured for different parameters, namely soil pH (soil:water ratio 1:2) using a pH-meter with an adjusted glass electrode (FE20, Mettler-Toledo Instruments), Soil texture was determined using Microtrac S3500 instrument based on the laser diffraction method according to the manufacturer's instructions, total organic carbon (TOC) based on the chromic acid wet oxidation method (Walkley and Black, 1933), nitrate ($N\text{-}NO_3$) and nitrite ($N\text{-}NH_4$) using the spectrophotometric determination method (Norman and Stucki, 1981), total soil phosphorus (TP) by molybdenum blue method (Murphy and Riley, 1962) and the soil chemical elements K_2O, Na_2O, MgO, Cl^-, Cu^{2+}, Mn^{2+}, Fe^{2+} and Zn^{2+} using the atomic absorption method (Lindsay and Norvell, 1978)

Bouyoucos, G. J. (2010). Hydrometer method improved for making particle size analyses of soils. *Agron. J.* 54, 464. doi: 10.2134/agronj1962.00021962005400050028x.

Lindsay, W. L., and Norvell, W. A. (1978). Development of a DTPA soil test for zinc, iron, manganese, and copper. *Soil Sci. Soc. Am. J.* 42, 421–428. doi: 10.2136/sssaj1978.03615995004200030009x.

Murphy, J., and Riley, J. (1962). A modified single solution method for the determination of phosphate in natural waters. *Anal. Chim. Acta* 27, 31–36. doi: 10.18393/ejss.477560.

Norman, R. J., and Stucki, J. W. (1981). The determination of nitrate and nitrite in soil extracts by ultraviolet spectrophotometry. *Soil Sci. Soc. Am. J.* 45, 347–353. doi: 10.2136/sssaj1981.03615995004500020024x.

Walkley, A., and Black, I. (1933). An examination of the Degtjareff method for determining soil organic matter, and a proposed modification of the chromic acid titration method. *Soil Sci* 37, 29–38.

2 Supplementary Figures and Tables

Supplementary Table S1. Results of ANOVA showing the significant effect of crop species identity (Ba, Dw, Cp, Fb), cropping system (AF, SC) and their interaction on dry matter production at flowering. AF, agroforestry system; SC, sole cropping system; Bs, bulk soil; Ba, barely; Dw, durum wheat; Cp, chickpeas; Fb, Faba bean. See also Fig. 2.

Factor	df	F	*P*
Species	3	41.88	< 0.0001 ***
System	1	59.04	< 0.0001 ***
Species × System	3	13.75	< 0.0001 ***

Supplementary Table S2. Nonparametric Spearman correlations used to find the associations between the biomass of soil procaryotic (a) and fungal (b) communities with the chemical composition of the soil. The canonical coefficients, the goodness-of-fit (R2), and the significance of the microbiome–environment relationship were assessed using the Monte Carlo permutation test (999 permutations).

(a)

ITS	cer	Leg
Axi1	28.13	96.34
AXi2	21.86	1.848
R2	0.9939	0.9879
R2adj	0.9904	0.981
F	286.1	143.1
Perm p, n=999	0.001	0.001

(b)

16S	Leg	Cer
Axi1	69.61	90.37
Axi2	22.9	7.297
R2	0.9944	0.983
R2 adj	0.9912	0.9734
F	309.4	101.5
Perm p, n=999	0.001	0.001

Supplementary Table S3. Quantitative report of taxa connection as a percentage (%) of the total degree for each phylum/class in procaryotic (a) and fungal (b) communities' networks of cereals and legumes in the agroforestry (AF) and sole Cropping (SC) systems.
ME, mutual exclusion (A mutual exclusion is a connection between micro-organisms that prevents simultaneous access to a shared resource);
CoP, co-presence (Co-presence is a connection between micro-organisms that facilitate simultaneous interaction to share resource)

**Edge interaction type%**	Bulk soil				Cereals								Legumes							
	Bs_AF		Bs_SC		Ba_AF		Ba_SC		Dw_AF		Dw_SC		Cp_AF		Cp_SC		Fb_AF		Fb_SC	
**(a) Prokaryotes**	ME	CoP	ME	CoP	ME	CoP	ME	CoP	ME	CoP	ME	CoP	ME	CoP	ME	CoP	ME	CoP	ME	CoP
Crenarchaeota	100	0	100	0	100	0	100	0	100	0	100	0	100	0	100	0	100	0	100	0
Euryarchaeota	0	0	0	0	100	0	100	0	0	0	0	0	100	0	0	0	0	0	0	0
Thermoplasmata	0	0	0	0	100	0	0	0	0	0	0	0	0	0	0	0	100	0	0	0
Acidobacteria	100	0	100	0	100	0	100	0	100	0	100	0	100	0	100	0	100	0	100	0
Actinobacteria	99	1	98	2	98	2	98	3	98	2	98	2	98	2	97	3	98	2	97	3
Armatimonadetes	0	0	0	0	0	0	0	0	100	0	0	0	0	0	0	0	100	0	0	0
Bacteroidetes	100	0	100	0	100	0	100	0	100	0	100	0	100	0	100	0	100	0	100	0
Alphaproteobacteria	100	0	100	0	100	0	100	0	100	0	100	0	100	0	100	0	100	0	100	0
Betaproteobacteria	100	0	100	0	100	0	100	0	100	0	100	0	100	0	100	0	100	0	100	0
Deltaproteobacteria	100	0	100	0	100	0	100	0	100	0	100	0	100	0	100	0	100	0	100	0
Gammaproteobacteria	100	0	100	0	100	0	100	0	100	0	100	0	100	0	100	0	100	0	100	0
Chloroflexi	100	0	100	0	100	0	100	0	100	0	100	0	100	0	100	0	100	0	100	0
Cyanobacteria	100	0	0	0	100	0	0	0	100	0	0	0	100	0	0	0	100	0	0	0
Fibrobacteres	0	0	0	0	0	0	0	0	0	0	0	0	0	0	0	0	0	0	100	0
Firmicutes	100	0	100	0	100	0	100	0	100	0	100	0	100	0	100	0	100	0	100	0
Gemmatimonadetes	100	0	100	0	100	0	100	0	100	0	100	0	100	0	100	0	100	0	100	0
Nitrospirae	100	0	0	0	100	0	100	0	100	0	100	0	100	0	100	0	100	0	100	0
Others	0	0	0	0	0	0	0	0	0	0	100	0	100	0	100	0	0	0	0	0
Planctomycetes	100	0	100	0	100	0	100	0	100	0	100	0	100	0	100	0	100	0	100	0
Verrucomicrobia	100	0	100	0	100	0	100	0	100	0	100	0	100	0	100	0	100	0	100	0
**(b) Fungi**																				
Agaricomycetes	93	7	91	9	91	9	87	13	79	21	93	7	93	7	79	21	95	5	97	3
Chytridiomycetes	0	0	0	0	0	100	100	0	0	0	0	0	100	0	100	0	80	20	100	0
Dothideomycetes	100	0	73	27	96	4	53	47	77	23	91	9	86	14	81	19	95	5	100	0
Eurotiomycetes	100	0	76	24	99	1	89	11	81	19	100	0	100	0	85	15	92	8	0	0
Exobasidiomycetes	0	0	0	0	100	0	0	0	100	0	0	0	0	0	100	0	0	0	0	0
Fibrobacteres	100	0	100	0	100	0	100	0	100	0	0	0	0	0	0	0	100	0	0	0
Glomeromycetes	100	0	100	0	100	0	11	89	100	0	100	0	100	0	100	0	100	0	0	100
Incertae_sedis	0	0	0	100	0	100	0	0	0	0	0	0	0	0	100	0	0	0	0	100
Lecanoromycetes	0	0	100	0	84	16	0	0	100	0	0	0	0	0	0	100	100	0	0	0
Leotiomycetes	0	0	100	0	100	0	0	0	100	0	0	0	100	0	100	0	100	0	0	0
Orbiliomycetes	0	0	0	0	0	0	0	0	100	0	0	0	100	0	100	0	100	0	0	0
Pezizomycetes	100	0	15	85	0	100	100	0	100	0	75	25	100	0	100	0	100	0	0	0
Saccharomycetes	0	0	0	0	0	0	0	100	0	0	100	0	0	0	0	0	0	0	0	0
Sordariomycetes	100	0	50	50	94	6	100	0	67	33	100	0	79	21	82	18	96	4	100	0

Ustilaginomycetes	0	0	0	0	100	0	100	0	0	0	0	0	100	0	0	0	0	0	0	0
Xylonomycetes	0	0	0	0	0	0	0	0	100	0	0	0	100	0	0	0	0	0	0	0
unidentified	40	60	62	38	0	0	100	0	100	0	100	0	100	0	75	25	100	0	50	50

Supplementary Table S4. Network topological characteristics calculated by network analyzer tool in Cytoscape v.3.8.

	**Bulk soil**		**Cereals**				**Legumes**			
	**Bs_AF**	**Bs_SC**	**Ba_AF**	**Ba_SC**	**Dw_AF**	**Dw_SC**	**Cp_AF**	**Cp_SC**	**Fb_AF**	**Fb_SC**
Number of nodes	151	200	242	198	206	181	224	196	297	136
Number of edges	249	470	545	360	433	317	463	415	584	141
Characteristic path length	4.785	5.389	6.155	4.525	5.433	4.613	4.858	6.006	3.686	5.433
Network density	0.027	0.0036	0.026	0.031	0.028	0.031	0.025	0.033	0.017	0.025
Network heterogeneity	1.119	1.105	1.259	1.133	1.142	1.094	1.237	1.121	2.788	1.021
Network centralization	0.17	1.139	0.141	0.228	0.109	0.12	0.145	0.135	0.506	0.184
Modularity	0.726	0.752	0.728	0.764	0.727	0.727	0.748	0.757	0.732	0.754
Number of communities	75	101	123	93	134	94	101	90	186	45

AF, Agroforestry; SC, sole cropping; Bs, bulk soil; Ba, barely; Dw, durum wheat; Cp, chickpeas; Fb, faba bean

Supplementary Table S5. HSD-Tukey test for comparison of the relative abundance of rhizospheric prokaryotic communities of cereals (barely and durum wheat) and legumes (chickpea and faba bean) crops in Agroforestry (AF) and sole cropping (SC) systems. Bs, bulk soil; Ba, barely; Dw, durum wheat; Cp, chickpeas; Fb, Faba bean

Dependent Variable	traitments		Mean Difference (I-J)	Std. Error	Sig.	95% Confidence Interval	
						Lower Bound	Upper Bound
**Crenarchaeota**	Bs_FS	Bs_AF	0.014239132	0.009923	0.901579	-0.020900063	0.04937833
		Ba_FS	0.012815218	0.009923	0.944414	-0.022323977	0.04795441
		Ba_AF	0.024918481	0.009923	0.319531	-0.010220714	0.06005768
		Dw_FS	-0.017086959	0.009923	0.771659	-0.052226154	0.01805224
		Dw_AF	0.008543479	0.009923	0.033869	0.001882548	0.07216094
		Fb_FS	0.008543479	0.009923	0.559004	-0.055785937	0.01449245
		Fb_AF	0.002821883	0.009923	0.002849	0.013273853	0.08355224
		Cp_FS	0.008543479	0.009923	0.559004	-0.055785937	0.01449245
		Cp_AF	0.012103262	0.009923	0.960179	-0.023035933	0.04724246
	Bs_AF	Bs_FS	-0.014239132	0.009923	0.901579	-0.049378327	0.02090006
		Ba_FS	-0.001423914	0.009923	1	-0.036563109	0.03371528
		Ba_AF	0.010679349	0.009923	0.981796	-0.024459846	0.04581854
		Dw_FS	-0.031326091	0.009923	0.106228	-0.066465286	0.0038131
		Dw_AF	0.022782611	0.009923	0.431748	-0.012356584	0.05792181
		Fb_FS	-0.034885874	0.009923	0.05265	-0.070025069	0.00025332
		Fb_AF	0.034173916	0.009923	0.06081	-0.000965279	0.06931311
		Cp_FS	-0.029190221	0.009923	0.157615	-0.064329416	0.00594897
		Cp_AF	-0.00213587	0.009923	1	-0.037275065	0.03300333
	Ba_FS	Bs_FS	-0.012815218	0.009923	0.944414	-0.047954413	0.02232398
		Bs_AF	0.001423914	0.009923	1	-0.033715281	0.03656311
		Ba_AF	0.012103262	0.009923	0.960179	-0.023035933	0.04724246
		Dw_FS	-0.029902177	0.009923	0.138551	-0.065041372	0.00523702
		Dw_AF	0.024206525	0.009923	0.354805	-0.01093267	0.05934572
		Fb_FS	-0.03346196	0.009923	0.070115	-0.068601155	0.00167724
		Fb_AF		0.009923	0.045514	0.000458635	0.07073703
		Cp_FS	-0.027766307	0.009923	0.202199	-0.062905502	0.00737289
		Cp_AF	-0.000711956	0.009923	1	-0.035851151	0.03442724
	Ba_AF	Bs_FS	-0.024918481	0.009923	0.319531	-0.060057676	0.01022071
		Bs_AF	-0.010679349	0.009923	0.981796	-0.045818544	0.02445985
		Ba_FS	-0.012103262	0.009923	0.960179	-0.047242457	0.02303593
		Dw_FS		0.009923	0.011654	-0.077144634	-0.0068662
		Dw_AF	0.012103263	0.009923	0.960179	-0.023035932	0.04724246
		Fb_FS		0.009923	0.005341	-0.080704417	-0.010426
		Fb_AF	0.023494568	0.009923	0.392278	-0.011644627	0.05863376
		Cp_FS		0.009923	0.018502	-0.075008764	-0.0047304
		Cp_AF	-0.012815219	0.009923	0.944414	-0.047954414	0.02232398

	Dw_FS	Bs_FS	0.017086959	0.009923	0.771659	-0.018052236	0.05222615
		Bs_AF	0.031326091	0.009923	0.106228	-0.003813104	0.06646529
		Ba_FS	0.029902177	0.009923	0.138551	-0.005237018	0.06504137
		Ba_AF		0.009923	0.011654	0.006866244	0.07714463
		Dw_AF		0.009923	0.000812	0.018969507	0.0892479
		Fb_FS	-0.003559783	0.009923	0.999997	-0.038698978	0.03157941
		Fb_AF		0.009923	7.05E-05	0.030360812	0.1006392
		Cp_FS	0.00213587	0.009923	1	-0.033003325	0.03727507
		Cp_AF	0.029190221	0.009923	0.157615	-0.005948974	0.06432942
	Dw_AF	Bs_FS		0.009923	0.033869	-0.072160938	-0.0018825
		Bs_AF	-0.022782611	0.009923	0.431748	-0.057921806	0.01235658
		Ba_FS	-0.024206525	0.009923	0.354805	-0.05934572	0.01093267
		Ba_AF	-0.012103263	0.009923	0.960179	-0.047242458	0.02303593
		Dw_FS		0.009923	0.000812	-0.089247897	-0.0189695
		Fb_FS		0.009923	0.000374	-0.09280768	-0.0225293
		Fb_AF	0.011391305	0.009923	0.97251	-0.02374789	0.0465305
		Cp_FS		0.009923	0.001299	-0.087112027	-0.0168336
		Cp_AF	-0.024918481	0.009923	0.319531	-0.060057676	0.01022071
	Fb_FS	Bs_FS	0.020646742	0.009923	0.559004	-0.014492453	0.05578594
		Bs_AF	0.034885874	0.009923	0.05265	-0.000253321	0.07002507
		Ba_FS	0.03346196	0.009923	0.070115	-0.001677235	0.06860116
		Ba_AF		0.009923	0.005341	0.010426027	0.08070442
		Dw_FS	0.003559783	0.009923	0.999997	-0.031579412	0.03869898
		Dw_AF		0.009923	0.000374	0.02252929	0.09280768
		Fb_AF	,09896*	0.022822	0.00932	0.018147218	0.17977672
		Cp_FS	0.005695653	0.009923	0.999836	-0.029443542	0.04083485
		Cp_AF	0.032750004	0.009923	0.080694	-0.002389191	0.0678892
	Fb_AF	Bs_FS		0.009923	0.002849	-0.083552243	-0.0132739
		Bs_AF	-0.034173916	0.009923	0.06081	-0.069313111	0.00096528
		Ba_FS		0.009923	0.045514	-0.070737025	-0.0004586
		Ba_AF	-0.023494568	0.009923	0.392278	-0.058633763	0.01164463
		Dw_FS		0.009923	7.05E-05	-0.100639202	-0.0303608
		Dw_AF	-0.011391305	0.009923	0.97251	-0.0465305	0.02374789
		Fb_FS	,09896*	0.022822	0.00932	0.018147218	0.17977672
		Cp_FS		0.009923	0.00011	-0.098503332	-0.0282249
		Cp_AF		0.009923	0.039288	-0.071448981	-0.0011706
	Cp_FS	Bs_FS	0.014951089	0.009923	0.874358	-0.020188106	0.05009028
		Bs_AF	0.029190221	0.009923	0.157615	-0.005948974	0.06432942
		Ba_FS	0.027766307	0.009923	0.202199	-0.007372888	0.0629055
		Ba_AF	0.008543479	0.009923	0.018502	0.004730374	0.07500876
		Dw_FS	-0.00213587	0.009923	1	-0.037275065	0.03300333
		Dw_AF		0.009923	0.001299	0.016833637	0.08711203
		Fb_FS	-0.005695653	0.009923	0.999836	-0.040834848	0.02944354
		Fb_AF		0.009923	0.00011	0.028224942	0.09850333

		Cp_AF	0.027054351	0.009923	0.227929	-0.008084844	0.06219355
	Cp_AF	Bs_FS	-0.012103262	0.009923	0.960179	-0.047242457	0.02303593
		Bs_AF	0.00213587	0.009923	1	-0.033003325	0.03727507
		Ba_FS	0.000711956	0.009923	1	-0.034427239	0.03585115
		Ba_AF	0.012815219	0.009923	0.944414	-0.022323976	0.04795441
		Dw_FS	-0.029190221	0.009923	0.157615	-0.064329416	0.00594897
		Dw_AF	0.024918481	0.009923	0.319531	-0.010220714	0.06005768
		Fb_FS	-0.032750004	0.009923	0.080694	-0.067889199	0.00238919
		Fb_AF		0.009923	0.039288	0.001170591	0.07144898
		Cp_FS	-0.027054351	0.009923	0.227929	-0.062193546	0.00808484
Bacteroidetes	Bs_FS	Bs_AF		0.001884	1.02E-05	0.007568889	0.02090938
		Ba_FS		0.001884	0.002639	0.002585193	0.01592568
		Ba_AF		0.001884	0.000508	0.004009106	0.01734959
		Dw_FS	0.00640761	0.001884	0.06613	-0.000262634	0.01307785
		Dw_AF		0.001884	2.39E-06	0.008992802	0.02233329
		Fb_FS	-0.000711956	0.001884	0.999995	-0.0073822	0.00595829
		Fb_AF		0.001884	2.16E-05	0.006856932	0.02019742
		Cp_FS	0.00427174	0.001884	0.447694	-0.002398504	0.01094198
		Cp_AF		0.001884	2.39E-06	0.008992802	0.02233329
	Bs_AF	Bs_FS		0.001884	1.02E-05	-0.020909376	-0.0075689
		Ba_FS	-0.004983696	0.001884	0.259694	-0.01165394	0.00168655
		Ba_AF	-0.003559783	0.001884	0.67538	-0.010230027	0.00311046
		Dw_FS		0.001884	0.013713	-0.014501766	-0.0011613
		Dw_AF	0.001423913	0.001884	0.998537	-0.00524633	0.00809416
		Fb_FS		0.001884	4.89E-06	-0.021621332	-0.0082808
		Fb_AF	-0.000711957	0.001884	0.999995	-0.0073822	0.00595829
		Cp_FS		0.001884	0.001154	-0.016637636	-0.0032971
		Cp_AF	0.001423913	0.001884	0.998537	-0.00524633	0.00809416
	Ba_FS	Bs_FS		0.001884	0.002639	-0.015925679	-0.0025852
		Bs_AF	-,29119*	0.043143	5.19E-05	-0.443965108	0.01165394
		Ba_AF	0.001423913	0.001884	0.998537	-0.00524633	0.00809416
		Dw_FS	-0.002847826	0.001884	0.872246	-0.00951807	0.00382242
		Dw_AF	0.006407609	0.001884	0.06613	-0.000262634	0.01307785
		Fb_FS		0.001884	0.001154	-0.016637636	-0.0032971
		Fb_AF	0.00427174	0.001884	0.447694	-0.002398504	0.01094198
		Cp_FS	-0.004983696	0.001884	0.259694	-0.01165394	0.00168655
		Cp_AF	0.006407609	0.001884	0.06613	-0.000262634	0.01307785
	Ba_AF	Bs_FS		0.001884	0.000508	-0.017349592	-0.0040091
		Bs_AF	0.003559783	0.001884	0.67538	-0.00311046	0.01023003
		Ba_FS	-,29119*	0.043143	5.19E-05	-0.443965108	0.00524633
		Dw_FS	-0.004271739	0.001884	0.447694	-0.010941983	0.0023985
		Dw_AF	0.004983696	0.001884	0.259694	-0.001686547	0.01165394
		Fb_FS		0.001884	0.000226	-0.018061549	-0.0047211
		Fb_AF	0.002847827	0.001884	0.872246	-0.003822417	0.00951807

		Cp_FS	-0.006407609	0.001884	0.06613	-0.013077853	0.00026263
		Cp_AF	0.004983696	0.001884	0.259694	-0.001686547	0.01165394
	Dw_FS	Bs_FS	-0.00640761	0.001884	0.06613	-0.013077853	0.00026263
		Bs_AF		0.001884	0.013713	0.001161279	0.01450177
		Ba_FS	0.002847826	0.001884	0.872246	-0.003822417	0.00951807
		Ba_AF	0.004271739	0.001884	0.447694	-0.002398504	0.01094198
		Dw_AF		0.001884	0.002639	0.002585192	0.01592568
		Fb_FS		0.001884	0.030594	-0.013789809	-0.0004493
		Fb_AF		0.001884	0.030594	0.000449323	0.01378981
		Cp_FS	-0.00213587	0.001884	0.974554	-0.008806113	0.00453437
		Cp_AF		0.001884	0.002639	0.002585192	0.01592568
	Dw_AF	Bs_FS		0.001884	2.39E-06	-0.022333289	-0.0089928
		Bs_AF	-0.001423913	0.001884	0.998537	-0.008094156	0.00524633
		Ba_FS	-0.006407609	0.001884	0.06613	-0.013077853	0.00026263
		Ba_AF	-0.004983696	0.001884	0.259694	-0.01165394	0.00168655
		Dw_FS		0.001884	0.002639	-0.015925679	-0.0025852
		Fb_FS		0.001884	1.19E-06	-0.023045245	-0.0097048
		Fb_AF	-0.00213587	0.001884	0.974554	-0.008806113	0.00453437
		Cp_FS		0.001884	0.000226	-0.018061549	-0.0047211
		Cp_AF	0	0.001884	1	-0.006670243	0.00667024
	Fb_FS	Bs_FS	0.000711956	0.001884	0.999995	-0.005958287	0.0073822
		Bs_AF		0.001884	4.89E-06	0.008280845	0.02162133
		Ba_FS		0.001884	0.001154	0.003297149	0.01663764
		Ba_AF		0.001884	0.000226	0.004721062	0.01806155
		Dw_FS		0.001884	0.030594	0.000449323	0.01378981
		Dw_AF		0.001884	1.19E-06	0.009704758	0.02304524
		Fb_AF		0.001884	1.02E-05	0.007568889	0.02090938
		Cp_FS	0.004983696	0.001884	0.259694	-0.001686547	0.01165394
		Cp_AF		0.001884	1.19E-06	0.009704758	0.02304524
	Fb_AF	Bs_FS		0.001884	2.16E-05	-0.020197419	-0.0068569
		Bs_AF	0.000711957	0.001884	0.999995	-0.005958287	0.0073822
		Ba_FS	-0.00427174	0.001884	0.447694	-0.010941983	0.0023985
		Ba_AF	-0.002847827	0.001884	0.872246	-0.00951807	0.00382242
		Dw_FS		0.001884	0.030594	-0.013789809	-0.0004493
		Dw_AF	0.00213587	0.001884	0.974554	-0.004534374	0.00880611
		Fb_FS		0.001884	1.02E-05	-0.020909375	-0.0075689
		Cp_FS		0.001884	0.002639	-0.015925679	-0.0025852
		Cp_AF	0.00213587	0.001884	0.974554	-0.004534374	0.00880611
	Cp_FS	Bs_FS	-0.00427174	0.001884	0.447694	-0.010941983	0.0023985
		Bs_AF		0.001884	0.001154	0.003297149	0.01663764
		Ba_FS	0.004983696	0.001884	0.259694	-0.001686547	0.01165394
		Ba_AF	0.006407609	0.001884	0.06613	-0.000262634	0.01307785
		Dw_FS	0.00213587	0.001884	0.974554	-0.004534373	0.00880611
		Dw_AF		0.001884	0.000226	0.004721062	0.01806155

		Fb_FS	-0.004983696	0.001884	0.259694	-0.011653939	0.00168655
		Fb_AF		0.001884	0.002639	0.002585193	0.01592568
		Cp_AF		0.001884	0.000226	0.004721062	0.01806155
	Cp_AF	Bs_FS		0.001884	2.39E-06	-0.022333289	-0.0089928
		Bs_AF	-0.001423913	0.001884	0.998537	-0.008094156	0.00524633
		Ba_FS	-0.006407609	0.001884	0.06613	-0.013077853	0.00026263
		Ba_AF	-0.004983696	0.001884	0.259694	-0.01165394	0.00168655
		Dw_FS		0.001884	0.002639	-0.015925679	-0.0025852
		Dw_AF	0	0.001884	1	-0.006670243	0.00667024
		Fb_FS		0.001884	1.19E-06	-0.023045245	-0.0097048
		Fb_AF	-0.00213587	0.001884	0.974554	-0.008806113	0.00453437
		Cp_FS		0.001884	0.000226	-0.018061549	-0.0047211
Planctomycetes	Bs_FS	Bs_AF		0.002733	8.02E-09	0.022360993	0.0417151
		Ba_FS	-0.005695653	0.002733	0.556836	-0.015372707	0.0039814
		Ba_AF		0.002733	5.46E-09	0.023072949	0.04242706
		Dw_FS		0.002733	0.000254	0.006697947	0.02605206
		Dw_AF		0.002733	1.09E-12	-0.162747723	-0.1433936
		Fb_FS	-0.00213587	0.002733	0.998113	-0.011812924	0.00754118
		Fb_AF		0.002733	2.65E-08	0.020225123	0.03957923
		Cp_FS		0.002733	4.93E-05	0.008833817	0.02818793
		Cp_AF		0.002733	9.34E-08	0.018089253	0.03744336
	Bs_AF	Bs_FS		0.002733	8.02E-09	-0.041715102	-0.022361
		Ba_FS		0.002733	4.35E-10	-0.047410755	-0.0280566
		Ba_AF	0.000711957	0.002733	1	-0.008965098	0.01038901
		Dw_FS		0.002733	0.000444	-0.0253401	-0.005986
		Dw_AF		0.002733	1.09E-12	-0.194785771	-0.1754317
		Fb_FS		0.002733	2.57E-09	-0.043850972	-0.0244969
		Fb_AF	-0.00213587	0.002733	0.998113	-0.011812925	0.00754118
		Cp_FS		0.002733	0.002437	-0.02320423	-0.0038501
		Cp_AF	-0.00427174	0.002733	0.850303	-0.013948794	0.00540531
	Ba_FS	Bs_FS	0.005695653	0.002733	0.556836	-0.003981402	0.01537271
		Bs_AF		0.002733	4.35E-10	0.028056645	0.04741075
		Ba_AF		0.002733	3.1E-10	0.028768602	0.04812271
		Dw_FS		0.002733	3.75E-06	0.0123936	0.03174771
		Dw_AF		0.002733	1.09E-12	-0.157052071	-0.137698
		Fb_FS	0.003559783	0.002733	0.941626	-0.006117272	0.01323684
		Fb_AF		0.002733	1.24E-09	0.025920775	0.04527488
		Cp_FS		0.002733	8.84E-07	0.01452947	0.03388358
		Cp_AF		0.002733	3.73E-09	0.023784906	0.04313901
	Ba_AF	Bs_FS		0.002733	5.46E-09	-0.042427059	-0.0230729
		Bs_AF	-0.000711957	0.002733	1	-0.010389011	0.0089651
		Ba_FS		0.002733	3.1E-10	-0.048122711	-0.0287686
		Dw_FS		0.002733	0.000254	-0.026052057	-0.0066979
		Dw_AF		0.002733	1.09E-12	-0.195497727	-0.1761436

		Fb_FS		0.002733	1.78E-09	-0.044562928	-0.0252088
		Fb_AF	-0.002847827	0.002733	0.985275	-0.012524881	0.00682923
		Cp_FS		0.002733	0.001378	-0.023916187	-0.0045621
		Cp_AF	-0.004983696	0.002733	0.714471	-0.014660751	0.00469336
	Dw_FS	Bs_FS		0.002733	0.000254	-0.026052057	-0.0066979
		Bs_AF		0.002733	0.000444	0.005985991	0.0253401
		Ba_FS		0.002733	3.75E-06	-0.031747709	-0.0123936
		Ba_AF		0.002733	0.000254	0.006697947	0.02605206
		Dw_AF	-2,35658*	0.086221	1.09E-12	-2.661894376	-2.0512583
		Fb_FS		0.002733	4.93E-05	-0.028187926	-0.0088338
		Fb_AF		0.002733	0.002437	0.003850121	0.02320423
		Cp_FS	0.00213587	0.002733	0.998113	-0.007541185	0.01181292
		Cp_AF		0.002733	0.013398	0.001714251	0.02106836
	Dw_AF	Bs_FS		0.002733	1.09E-12	0.143393614	0.16274772
		Bs_AF		0.002733	1.09E-12	0.175431661	0.19478577
		Ba_FS		0.002733	1.09E-12	0.137697961	0.15705207
		Ba_AF		0.002733	1.09E-12	0.176143618	0.19549773
		Dw_FS	-2,35658*	0.086221	1.09E-12	-2.661894376	-2.0512583
		Fb_FS		0.002733	1.09E-12	0.141257744	0.16061185
		Fb_AF		0.002733	1.09E-12	0.173295791	0.1926499
		Cp_FS		0.002733	1.09E-12	0.161904486	0.1812586
		Cp_AF		0.002733	1.09E-12	0.171159922	0.19051403
	Fb_FS	Bs_FS	0.00213587	0.002733	0.998113	-0.007541185	0.01181292
		Bs_AF		0.002733	2.57E-09	0.024496862	0.04385097
		Ba_FS	-0.003559783	0.002733	0.941626	-0.013236838	0.00611727
		Ba_AF		0.002733	1.78E-09	0.025208819	0.04456293
		Dw_FS		0.002733	4.93E-05	0.008833817	0.02818793
		Dw_AF		0.002733	1.09E-12	-0.160611854	-0.1412577
		Fb_AF		0.002733	8.02E-09	0.022360992	0.0417151
		Cp_FS		0.002733	1.03E-05	0.010969687	0.0303238
		Cp_AF		0.002733	2.65E-08	0.020225123	0.03957923
	Fb_AF	Bs_FS		0.002733	2.65E-08	-0.039579232	-0.0202251
		Bs_AF	0.00213587	0.002733	0.998113	-0.007541185	0.01181292
		Ba_FS		0.002733	1.24E-09	-0.045274885	-0.0259208
		Ba_AF	0.002847827	0.002733	0.985275	-0.006829228	0.01252488
		Dw_FS		0.002733	0.002437	-0.02320423	-0.0038501
		Dw_AF		0.002733	1.09E-12	-0.192649901	-0.1732958
		Fb_FS		0.002733	8.02E-09	-0.041715102	-0.022361
		Cp_FS		0.002733	0.013398	-0.02106836	-0.0017143
		Cp_AF	-0.00213587	0.002733	0.998113	-0.011812924	0.00754118
	Cp_FS	Bs_FS		0.002733	4.93E-05	-0.028187927	-0.0088338
		Bs_AF		0.002733	0.002437	0.003850121	0.02320423
		Ba_FS		0.002733	8.84E-07	-0.033883579	-0.0145295
		Ba_AF		0.002733	0.001378	0.004562077	0.02391619

		Dw_FS	-0.00213587	0.002733	0.998113	-0.011812925	0.00754118
		Dw_AF		0.002733	1.09E-12	-0.181258595	-0.1619045
		Fb_FS		0.002733	1.03E-05	-0.030323796	-0.0109697
		Fb_AF		0.002733	0.013398	0.001714251	0.02106836
		Cp_AF	0.009255436	0.002733	0.068104	-0.000421619	0.01893249
	Cp_AF	Bs_FS		0.002733	9.34E-08	-0.037443362	-0.0180893
		Bs_AF	0.00427174	0.002733	0.850303	-0.005405315	0.01394879
		Ba_FS		0.002733	3.73E-09	-0.043139015	-0.0237849
		Ba_AF	0.004983696	0.002733	0.714471	-0.004693358	0.01466075
		Dw_FS		0.002733	0.013398	-0.02106836	-0.0017143
		Dw_AF		0.002733	1.09E-12	-0.190514031	-0.1711599
		Fb_FS		0.002733	2.65E-08	-0.039579232	-0.0202251
		Fb_AF	0.00213587	0.002733	0.998113	-0.007541185	0.01181292
		Cp_FS	-0.009255436	0.002733	0.068104	-0.01893249	0.00042162
Alphaproteobacteria	Bs_SC	Bs_AF		0.086221	1.09E-12	1.891780034	2.5024161
		Ba_SC		0.086221	2.32E-06	-1.023682239	-0.4130462
		Ba_AF		0.086221	1.09E-12	3.511481294	4.12211736
		Dw_SC		0.086221	1.09E-12	1.841943074	2.45257914
		Dw_AF		0.086221	1.09E-12	7.0612969	7.67193297
		Fb_SC		0.086221	1.09E-12	2.701986644	3.31262271
		Fb_AF		0.086221	0.001711	-0.746019166	-0.1353831
		Cp_SC		0.086221	1.014593	0.050660267	0.66129633
		Cp_AF		0.086221	1.09E-12	2.051258311	2.66189438
	Bs_AF	Bs_SC		0.086221	1.09E-12	-2.502416099	-1.89178
		Ba_SC		0.086221	1.09E-12	-3.220780306	-2.6101442
		Ba_AF		0.086221	2.41E-12	1.314383227	1.92501929
		Dw_SC	-0.04983696	0.086221	0.999826	-0.355154993	0.25548107
		Dw_AF		0.086221	1.09E-12	4.864198833	5.4748349
		Fb_SC		0.086221	3.41E-07	0.504888577	1.11552464
		Fb_AF		0.086221	1.09E-12	-2.943117233	-2.3324812
		Cp_SC		0.086221	1.2E-12	-2.146437799	-1.5358017
		Cp_AF	0.159478277	0.086221	0.699268	-0.145839756	0.46479631
	Ba_SC	Bs_SC		0.086221	2.32E-06	0.413046174	1.02368224
		Bs_AF		0.086221	1.09E-12	2.610144241	3.22078031
		Ba_AF		0.086221	1.09E-12	4.229845501	4.84048157
		Dw_SC		0.086221	1.09E-12	2.560307281	3.17094335
		Dw_AF		0.086221	1.09E-12	7.779661107	8.39029717
		Fb_SC		0.086221	1.09E-12	3.420350851	4.03098692
		Fb_AF	0.277663073	0.086221	0.094157	-0.027654959	0.58298111
		Cp_SC		0.086221	2.74E-09	0.769024474	1.37966054
		Cp_AF		0.086221	1.09E-12	2.769622517	3.38025858
	Ba_AF	Bs_SC		0.086221	1.09E-12	-4.122117359	-3.5114813
		Bs_AF		0.086221	2.41E-12	-1.925019293	-1.3143832
		Ba_SC		0.086221	1.09E-12	-4.840481566	-4.2298455

		Dw_SC		0.086221	1.81E-12	-1.974856253	-1.3642202
		Dw_AF		0.086221	1.09E-12	3.244497573	3.85513364
		Fb_SC		0.086221	3.46E-07	-1.114812683	-0.5041766
		Fb_AF		0.086221	1.09E-12	-4.562818493	-3.9521824
		Cp_SC		0.086221	1.09E-12	-3.766139059	-3.155503
		Cp_AF		0.086221	1.1E-11	-1.765541016	-1.154905
	Dw_SC	Bs_SC		0.086221	1.09E-12	-2.452579139	-1.8419431
		Bs_AF	0.04983696	0.086221	0.999826	-0.255481073	0.35515499
		Ba_SC		0.086221	1.09E-12	-3.170943346	-2.5603073
		Ba_AF		0.086221	1.81E-12	1.364220187	1.97485625
		Dw_AF		0.086221	1.09E-12	4.914035793	5.52467186
		Fb_SC		0.086221	1.27E-07	0.554725537	1.1653616
		Fb_AF		0.086221	1.09E-12	-2.893280273	-2.2826442
		Cp_SC		0.086221	1.27E-12	-2.096600839	-1.4859648
		Cp_AF	0.209315237	0.086221	0.360782	-0.096002796	0.51463327
	Dw_AF	Bs_SC		0.086221	1.09E-12	-7.671932965	-7.0612969
		Bs_AF		0.086221	1.09E-12	-5.474834899	-4.8641988
		Ba_SC		0.086221	1.09E-12	-8.390297172	-7.7796611
		Ba_AF		0.086221	1.09E-12	-3.855133639	-3.2444976
		Dw_SC		0.086221	1.09E-12	-5.524671859	-4.9140358
		Fb_SC		0.086221	1.09E-12	-4.664628289	-4.0539922
		Fb_AF		0.086221	1.09E-12	-8.112634099	-7.501998
		Cp_SC		0.086221	1.09E-12	-7.315954665	-6.7053186
		Cp_AF		0.086221	1.09E-12	-5.315356622	-4.7047206
	Fb_SC	Bs_SC		0.086221	1.09E-12	-3.312622709	-2.7019866
		Bs_AF		0.086221	3.41E-07	-1.115524643	-0.5048886
		Ba_SC		0.086221	1.09E-12	-4.030986916	-3.4203509
		Ba_AF		0.086221	3.46E-07	0.504176617	1.11481268
		Dw_SC		0.086221	1.27E-07	-1.165361603	-0.5547255
		Dw_AF		0.086221	1.09E-12	4.053992223	4.66462829
		Fb_AF	-,79668*	0.086221	4.48E-07	-1.101997466	-0.4913614
		Cp_SC		0.086221	1.09E-12	-2.956644409	-2.3460083
		Cp_AF		0.086221	1.04E-05	-0.956046366	-0.3454103
	Fb_AF	Bs_SC		0.086221	0.001711	0.135383101	0.74601917
		Bs_AF		0.086221	1.09E-12	2.332481167	2.94311723
		Ba_SC	-0.277663073	0.086221	0.094157	-0.582981106	0.02765496
		Ba_AF		0.086221	1.09E-12	3.952182427	4.56281849
		Dw_SC		0.086221	1.09E-12	2.282644207	2.89328027
		Dw_AF		0.086221	1.09E-12	7.501998033	8.1126341
		Fb_SC	-,79668*	0.086221	4.48E-07	-1.101997466	-0.4913614
		Cp_SC		0.086221	1.09E-12	0.491361401	1.10199747
		Cp_AF		0.086221	1.09E-12	2.491959444	3.10259551
	Cp_SC	Bs_SC		0.086221	0.014593	-0.661296333	-0.0506603
		Bs_AF		0.086221	1.2E-12	1.535801734	2.1464378

		Ba_SC		0.086221	2.74E-09	-1.379660539	-0.7690245
		Ba_AF		0.086221	1.09E-12	3.155502994	3.76613906
		Dw_SC		0.086221	1.27E-12	1.485964774	2.09660084
		Dw_AF		0.086221	1.09E-12	6.7053186	7.31595467
		Fb_SC		0.086221	1.09E-12	2.346008344	2.95664441
		Fb_AF		0.086221	1.09E-12	-1.101997466	-0.4913614
		Cp_AF		0.086221	1.1E-12	1.695280011	2.30591608
	Cp_AF	Bs_SC		0.086221	1.09E-12	-2.661894376	-2.0512583
		Bs_AF	-0.159478277	0.086221	0.699268	-0.464796309	0.14583976
		Ba_SC		0.086221	1.09E-12	-3.380258583	-2.7696225
		Ba_AF		0.086221	1.1E-11	1.154904951	1.76554102
		Dw_SC	-0.209315237	0.086221	0.360782	-0.514633269	0.0960028
		Dw_AF		0.086221	1.09E-12	4.704720557	5.31535662
		Fb_SC		0.086221	1.04E-05	0.345410301	0.95604637
		Fb_AF		0.086221	1.09E-12	-3.102595509	-2.4919594
		Cp_SC		0.086221	1.1E-12	-2.305916076	-1.69528
Deltaproteobacteria	Bs_SC	Bs_AF	0.041293483	0.043143	0.991749	-0.111481376	0.19406834
		Ba_SC		0.043143	7.26E-08	-0.597747734	-0.292198
		Ba_AF		0.043143	1.28E-05	-0.473867285	-0.1683176
		Dw_SC	-0.13811958	0.043143	0.097624	-0.290894439	0.01465528
		Dw_AF		0.043143	1.09E-12	-12.75440666	-12.448857
		Fb_SC	0.103233707	0.043143	0.378888	-0.049541152	0.25600857
		Fb_AF		0.043143	2.51E-11	-0.84978037	-0.5442307
		Cp_SC		0.043143	2.56E-09	-0.692437961	-0.3868882
		Cp_AF	-0.029902177	0.043143	0.999253	-0.182677036	0.12287268
	Bs_AF	Bs_SC	-0.041293483	0.043143	0.991749	-0.194068342	0.11148138
		Ba_SC		0.043143	1.59E-08	-0.639041216	-0.3334915
		Ba_AF		0.043143	2.05E-06	-0.515160768	-0.209611
		Dw_SC		0.043143	0.013685	-0.332187922	-0.0266382
		Dw_AF		0.043143	1.09E-12	-12.79570015	-12.49015
		Fb_SC	0.061940224	0.043143	0.901312	-0.090834635	0.21471508
		Fb_AF		0.043143	9.19E-12	-0.891073852	-0.5855241
		Cp_SC		0.043143	6.84E-10	-0.733731444	-0.4281817
		Cp_AF	-0.07119566	0.043143	0.809023	-0.223970519	0.0815792
	Ba_SC	Bs_SC		0.043143	7.26E-08	0.292198016	0.59774773
		Bs_AF		0.043143	1.59E-08	0.333491498	0.63904122
		Ba_AF	0.123880449	0.043143	0.178322	-0.02889441	0.27665531
		Dw_SC		0.043143	2.48E-05	0.154078435	0.45962815
		Dw_AF		0.043143	1.09E-12	-12.30943379	-12.003884
		Fb_SC		0.043143	1.94E-09	0.395431723	0.70098144
		Fb_AF		0.043143	0.00035	-0.404807495	-0.0992578
		Cp_SC	-0.094690227	0.043143	0.49021	-0.247465086	0.05808463
		Cp_AF		0.043143	2.31E-07	0.262295839	0.56784556
	Ba_AF	Bs_SC		0.043143	1.28E-05	0.168317567	0.47386729

		Bs_AF		0.043143	2.05E-06	0.20961105	0.51516077
		Ba_SC	-0.123880449	0.043143	0.178322	-0.276655308	0.02889441
		Dw_SC		0.043143	0.011454	0.030197987	0.3357477
		Dw_AF		0.043143	1.09E-12	-12.43331424	-12.127765
		Fb_SC		0.043143	1.61E-07	0.271551274	0.57710099
		Fb_AF		0.043143	1.15E-06	-0.528687944	-0.2231382
		Cp_SC		0.043143	0.001889	-0.371345535	-0.0657958
		Cp_AF		0.043143	0.001889	0.13841539	0.44396511
	Dw_SC	Bs_SC	0.13811958	0.043143	0.097624	-0.014655279	0.29089444
		Bs_AF		0.043143	0.013685	0.026638204	0.33218792
		Ba_SC		0.043143	2.48E-05	-0.459628153	-0.1540784
		Ba_AF		0.043143	0.011454	-0.335747705	-0.030198
		Dw_AF	,79668*	0.086221	4.48E-07	0.491361401	1.10199747
		Fb_SC		0.043143	0.000598	0.088578428	0.39412815
		Fb_AF		0.043143	1.37E-09	-0.711660789	-0.4061111
		Cp_SC		0.043143	3.98E-07	-0.554318381	-0.2487687
		Cp_AF	0.108217403	0.043143	0.320865	-0.044557456	0.26099226
	Dw_AF	Bs_SC		0.043143	1.09E-12	12.44885695	12.7544067
		Bs_AF		0.043143	1.09E-12	12.49015043	12.7957001
		Ba_SC		0.043143	1.09E-12	12.00388407	12.3094338
		Ba_AF		0.043143	1.09E-12	12.12776452	12.4333142
		Dw_SC	,79668*	0.086221	4.48E-07	0.491361401	1.10199747
		Fb_SC		0.043143	1.09E-12	12.55209065	12.8576404
		Fb_AF		0.043143	1.09E-12	11.75185144	12.0574012
		Cp_SC		0.043143	1.09E-12	11.90919384	12.2147436
		Cp_AF		0.043143	1.09E-12	12.41895477	12.7245045
	Fb_SC	Bs_SC	-0.103233707	0.043143	0.378888	-0.256008566	0.04954115
		Bs_AF	-0.061940224	0.043143	0.901312	-0.214715083	0.09083463
		Ba_SC		0.043143	1.94E-09	-0.700981441	-0.3954317
		Ba_AF		0.043143	1.61E-07	-0.577100992	-0.2715513
		Dw_SC		0.043143	0.000598	-0.394128146	-0.0885784
		Dw_AF		0.043143	1.09E-12	-12.85764037	-12.552091
		Fb_AF		0.043143	2.79E-12	-0.953014077	-0.6474644
		Cp_SC		0.043143	1.09E-10	-0.795671668	-0.4901219
		Cp_AF	-0.133135884	0.043143	0.121288	-0.285910743	0.01963898
	Fb_AF	Bs_SC		0.043143	2.51E-11	0.544230652	0.84978037
		Bs_AF		0.043143	9.19E-12	0.585524134	0.89107385
		Ba_SC		0.043143	0.00035	0.099257777	0.4048075
		Ba_AF		0.043143	1.15E-06	0.223138226	0.52868794
		Dw_SC		0.043143	1.37E-09	0.406111071	0.71166079
		Dw_AF		0.043143	1.09E-12	-12.05740115	-11.751851
		Fb_SC		0.043143	2.79E-12	0.647464359	0.95301408
		Cp_SC		0.043143	0.040279	0.00456755	0.31011727
		Cp_AF		0.043143	5.55E-11	0.514328475	0.81987819

	Cp_SC	Bs_SC		0.043143	2.56E-09	0.386888243	0.69243796
		Bs_AF		0.043143	6.84E-10	0.428181726	0.73373144
		Ba_SC	0.094690227	0.043143	0.49021	-0.058084632	0.24746509
		Ba_AF		0.043143	0.001889	0.065795817	0.37134554
		Dw_SC		0.043143	3.98E-07	0.248768663	0.55431838
		Dw_AF		0.043143	1.09E-12	-12.21474356	-11.909194
		Fb_SC		0.043143	1.09E-10	0.49012195	0.79567167
		Fb_AF		0.043143	0.040279	-0.310117268	-0.0045675
		Cp_AF		0.043143	7E-09	0.356986066	0.66253578
	Cp_AF	Bs_SC	0.029902177	0.043143	0.999253	-0.122872682	0.18267704
		Bs_AF	0.07119566	0.043143	0.809023	-0.081579199	0.22397052
		Ba_SC		0.043143	2.31E-07	-0.567845557	-0.2622958
		Ba_AF	-,29119*	0.043143	5.19E-05	-0.443965108	-0.1384154
		Dw_SC	-0.108217403	0.043143	0.320865	-0.260992262	0.04455746
		Dw_AF		0.043143	1.09E-12	-12.72450449	-12.418955
		Fb_SC	0.133135884	0.043143	0.121288	-0.019638975	0.28591074
		Fb_AF		0.043143	5.55E-11	-0.819878193	-0.5143285
		Cp_SC		0.043143	7E-09	-0.662535784	-0.3569861
Gammaproteobacteria	Bs_SC	Bs_AF		0.032839	1.09E-12	1.34607376	1.57864395
		Ba_SC		0.032839	2.88E-06	-0.386116645	-0.1535465
		Ba_AF		0.032839	1.14E-12	-0.838209086	-0.6056389
		Dw_SC		0.032839	3.23E-05	-0.345535119	-0.1129649
		Dw_AF		0.032839	4E-06	0.147850804	0.38042099
		Fb_SC		0.032839	1.81E-08	0.251084511	0.4836547
		Fb_AF		0.032839	1.1E-12	0.662595425	0.89516561
		Cp_SC		0.032839	1.11E-12	-0.87309496	-0.6405248
		Cp_AF		0.032839	1.09E-12	0.721687823	0.95425801
	Bs_AF	Bs_SC		0.032839	1.09E-12	-1.578643949	-1.3460738
		Ba_SC		0.032839	1.09E-12	-1.8484755	-1.6159053
		Ba_AF		0.032839	1.09E-12	-2.30056794	-2.0679978
		Dw_SC		0.032839	1.09E-12	-1.807893974	-1.5753238
		Dw_AF		0.032839	1.09E-12	-1.31450805	-1.0819379
		Fb_SC		0.032839	1.09E-12	-1.211274344	-0.9787042
		Fb_AF		0.032839	1.26E-12	-0.79976343	-0.5671932
		Cp_SC		0.032839	1.09E-12	-2.335453814	-2.1028836
		Cp_AF		0.032839	2.13E-12	-0.740671032	-0.5081008
	Ba_SC	Bs_SC		0.032839	2.88E-06	0.153546457	0.38611665
		Bs_AF		0.032839	1.09E-12	1.615905311	1.8484755
		Ba_AF	-1,10424*	0.076384	1.89E-10	-1.374727161	-0.8337622
		Dw_SC	0.040581526	0.032839	0.956953	-0.075703568	0.15686662
		Dw_AF		0.032839	2.24E-11	0.417682355	0.65025254
		Fb_SC		0.032839	1.78E-12	0.520916062	0.75348625
		Fb_AF		0.032839	1.09E-12	0.932426976	1.16499716
		Cp_SC		0.032839	1.19E-10	-0.603263409	-0.3706932

		Cp_AF		0.032839	1.09E-12	0.991519374	1.22408956
	Ba_AF	Bs_SC		0.032839	1.14E-12	0.605638897	0.83820909
		Bs_AF		0.032839	1.09E-12	2.067997752	2.30056794
		Ba_SC	-1,10424*	0.076384	1.89E-10	-1.374727161	-0.8337622
		Dw_SC		0.032839	9.62E-11	0.376388872	0.60895906
		Dw_AF		0.032839	1.09E-12	0.869774796	1.10234498
		Fb_SC		0.032839	1.09E-12	0.973008502	1.20557869
		Fb_AF		0.032839	1.09E-12	1.384519416	1.6170896
		Cp_SC	-0.034885874	0.032839	0.983277	-0.151170968	0.08139922
		Cp_AF		0.032839	1.09E-12	1.443611814	1.676182
	Dw_SC	Bs_SC		0.032839	3.23E-05	0.112964931	0.34553512
		Bs_AF		0.032839	1.09E-12	1.575323785	1.80789397
		Ba_SC	-0.040581526	0.032839	0.956953	-0.15686662	0.07570357
		Ba_AF		0.032839	9.62E-11	-0.608959061	-0.3763889
		Dw_AF		0.032839	9.37E-11	0.377100829	0.60967102
		Fb_SC		0.032839	3.63E-12	0.480334536	0.71290472
		Fb_AF		0.032839	1.09E-12	0.89184545	1.12441564
		Cp_SC		0.032839	2.78E-11	-0.643844935	-0.4112747
		Cp_AF		0.032839	1.09E-12	0.950937848	1.18350804
	Dw_AF	Bs_SC		0.032839	4E-06	-0.380420993	-0.1478508
		Bs_AF		0.032839	1.09E-12	1.081937862	1.31450805
		Ba_SC		0.032839	2.24E-11	-0.650252544	-0.4176824
		Ba_AF		0.032839	1.09E-12	-1.102344984	-0.8697748
		Dw_SC		0.032839	9.37E-11	-0.609671018	-0.3771008
		Fb_SC	0.103233707	0.032839	0.108896	-0.013051388	0.2195188
		Fb_AF		0.032839	4.33E-11	0.398459526	0.63102971
		Cp_SC		0.032839	1.09E-12	-1.137230858	-0.9046607
		Cp_AF		0.032839	6.5E-12	0.457551924	0.69012211
	Fb_SC	Bs_SC		0.032839	1.81E-08	-0.483654699	-0.2510845
		Bs_AF		0.032839	1.09E-12	0.978704155	1.21127434
		Ba_SC		0.032839	1.78E-12	-0.75348625	-0.5209161
		Ba_AF		0.032839	1.09E-12	-1.205578691	-0.9730085
		Dw_SC		0.032839	3.63E-12	-0.712904724	-0.4803345
		Dw_AF	-0.103233707	0.032839	0.108896	-0.219518801	0.01305139
		Fb_AF		0.032839	2.48E-09	0.29522582	0.52779601
		Cp_SC		0.032839	1.09E-12	-1.240464565	-1.0078944
		Cp_AF		0.032839	2.22E-10	0.354318218	0.58688841
	Fb_AF	Bs_SC		0.032839	1.1E-12	-0.895165613	-0.6625954
		Bs_AF		0.032839	1.26E-12	0.567193241	0.79976343
		Ba_SC		0.032839	1.09E-12	-1.164997164	-0.932427
		Ba_AF		0.032839	1.09E-12	-1.617089605	-1.3845194
		Dw_SC		0.032839	1.09E-12	-1.124415638	-0.8918454
		Dw_AF		0.032839	4.33E-11	-0.631029715	-0.3984595
		Fb_SC		0.032839	2.48E-09	-0.527796008	-0.2952258

		Cp_SC		0.032839	1.09E-12	-1.651975479	-1.4194053
		Cp_AF	0.059092398	0.032839	0.72844	-0.057192696	0.17537749
	Cp_SC	Bs_SC		0.032839	1.11E-12	0.640524771	0.87309496
		Bs_AF		0.032839	1.09E-12	2.102883625	2.33545381
		Ba_SC		0.032839	1.19E-10	0.37069322	0.60326341
		Ba_AF	0.034885874	0.032839	0.983277	-0.081399221	0.15117097
		Dw_SC		0.032839	2.78E-11	0.411274746	0.64384493
		Dw_AF		0.032839	1.09E-12	0.904660669	1.13723086
		Fb_SC		0.032839	1.09E-12	1.007894376	1.24046456
		Fb_AF		0.032839	1.09E-12	1.41940529	1.65197548
		Cp_AF		0.032839	1.09E-12	1.478497688	1.71106788
	Cp_AF	Bs_SC		0.032839	1.09E-12	0.954258011	-0.7216878
		Bs_AF		0.032839	2.13E-12	0.508100843	0.74067103
		Ba_SC		0.032839	1.09E-12	-1.224089562	-0.9915194
		Ba_AF		0.032839	1.09E-12	-1.676182003	-1.4436118
		Dw_SC		0.032839	1.09E-12	-1.183508036	-0.9509378
		Dw_AF		0.032839	6.5E-12	-0.690122113	-0.4575519
		Fb_SC		0.032839	2.22E-10	-0.586888406	-0.3543182
		Fb_AF	-0.059092398	0.032839	0.72844	-0.175377492	0.0571927
		Cp_SC		0.032839	1.09E-12	-1.711067877	-1.4784977
Verrucomicrobia	Bs_SC	Bs_AF		0.02475	1.09E-12	1.223070558	1.39835364
		Ba_SC		0.02475	9.43E-08	0.163679138	0.33896222
		Ba_AF		0.02475	1.28E-12	0.425679166	0.60096225
		Dw_SC		0.02475	2.68E-06	-0.291973085	-0.11669
		Dw_AF		0.02475	1.09E-12	0.764570508	0.93985359
		Fb_SC	-0.03559783	0.02475	0.900383	-0.123239371	0.05204371
		Fb_AF		0.02475	1.09E-12	1.102749892	1.27803297
		Cp_SC		0.02475	0.000117	0.069700867	0.24498395
		Cp_AF		0.02475	1.09E-12	1.131940113	1.30722319
	Bs_AF	Bs_SC		0.02475	1.09E-12	-1.39835364	-1.2230706
		Ba_SC		0.02475	1.09E-12	-1.147032961	-0.9717499
		Ba_AF		0.02475	1.09E-12	-0.885032932	-0.7097499
		Dw_SC		0.02475	1.09E-12	-1.602685184	-1.4274021
		Dw_AF		0.02475	2.83E-12	-0.546141591	-0.3708585
		Fb_SC		0.02475	1.09E-12	-1.43395147	-1.2586684
		Fb_AF		0.02475	0.002958	-0.207962207	-0.0326791
		Cp_SC		0.02475	1.09E-12	-1.241011232	-1.0657281
		Cp_AF		0.02475	0.037468	-0.178771986	-0.0034889
	Ba_SC	Bs_SC		0.02475	9.43E-08	-0.33896222	-0.1636791
		Bs_AF		0.02475	1.09E-12	0.971749879	1.14703296
		Ba_AF	1,10424*	0.076384	1.89E-10	0.033762209	1.37472716
		Dw_SC		0.02475	3.05E-12	-0.543293764	-0.3680107
		Dw_AF		0.02475	1.1E-12	0.513249829	0.68853291
		Fb_SC		0.02475	9.76E-09	-0.37456005	-0.199277

		Fb_AF		0.02475	1.09E-12	0.851429213	1.0267123
		Cp_SC		0.02475	0.029496	-0.181619812	-0.0063367
		Cp_AF		0.02475	1.09E-12	0.880619434	1.05590252
	Ba_AF	Bs_SC		0.02475	1.28E-12	-0.600962248	-0.4256792
		Bs_AF		0.02475	1.09E-12	0.70974985	0.88503293
		Ba_SC	1,10424*	0.076384	1.89E-10	0.033762209	1.37472716
		Dw_SC		0.02475	1.09E-12	-0.805293793	-0.6300107
		Dw_AF		0.02475	5.06E-10	0.251249801	0.42653288
		Fb_SC		0.02475	1.13E-12	-0.636560079	-0.461277
		Fb_AF		0.02475	1.09E-12	0.589429185	0.76471227
		Cp_SC		0.02475	2.07E-10	-0.44361984	-0.2683368
		Cp_AF		0.02475	1.09E-12	0.618619405	0.79390249
	Dw_SC	Bs_SC		0.02475	2.68E-06	0.116690003	0.29197309
		Bs_AF		0.02475	1.09E-12	1.427402102	1.60268518
		Ba_SC		0.02475	3.05E-12	0.368010682	0.54329376
		Ba_AF		0.02475	1.09E-12	0.630010711	0.80529379
		Dw_AF		0.02475	1.09E-12	0.968902052	1.14418513
		Fb_SC		0.02475	4.51E-05	0.081092173	0.25637526
		Fb_AF		0.02475	1.09E-12	1.307081436	1.48236452
		Cp_SC		0.02475	1.55E-10	0.274032411	0.44931549
		Cp_AF		0.02475	1.09E-12	1.336271657	1.51155474
	Dw_AF	Bs_SC		0.02475	1.09E-12	-0.93985359	-0.7645705
		Bs_AF		0.02475	2.83E-12	0.370858509	0.54614159
		Ba_SC		0.02475	1.1E-12	-0.688532911	-0.5132498
		Ba_AF		0.02475	5.06E-10	-0.426532883	-0.2512498
		Dw_SC		0.02475	1.09E-12	-1.144185134	-0.9689021
		Fb_SC		0.02475	1.09E-12	-0.97545142	-0.8001683
		Fb_AF		0.02475	5.26E-10	0.250537843	0.42582093
		Cp_SC		0.02475	1.09E-12	-0.782511182	-0.6072281
		Cp_AF		0.02475	1.17E-10	0.279728064	0.45501115
	Fb_SC	Bs_SC	0.03559783	0.02475	0.900383	-0.052043711	0.12323937
		Bs_AF		0.02475	1.09E-12	1.258668388	1.43395147
		Ba_SC		0.02475	9.76E-09	0.199276968	0.37456005
		Ba_AF		0.02475	1.13E-12	0.461276997	0.63656008
		Dw_SC		0.02475	4.51E-05	-0.256375255	-0.0810922
		Dw_AF		0.02475	1.09E-12	0.800168338	0.97545142
		Fb_AF		0.02475	1.09E-12	1.138347722	1.3136308
		Cp_SC		0.02475	6.43E-06	0.105298697	0.28058178
		Cp_AF		0.02475	1.09E-12	1.167537943	1.34282103
	Fb_AF	Bs_SC		0.02475	1.09E-12	-1.278032974	-1.1027499
		Bs_AF		0.02475	0.002958	0.032679125	0.20796221
		Ba_SC		0.02475	1.09E-12	-1.026712295	-0.8514292
		Ba_AF		0.02475	1.09E-12	-0.764712267	-0.5894292
		Dw_SC		0.02475	1.09E-12	-1.482364518	-1.3070814

		Dw_AF		0.02475	5.26E-10	-0.425820925	-0.2505378
		Fb_SC		0.02475	1.09E-12	-1.313630804	-1.1383477
		Cp_SC		0.02475	1.09E-12	-1.120690566	-0.9454075
		Cp_AF	0.029190221	0.02475	0.967501	-0.05845132	0.11683176
	Cp_SC	Bs_SC		0.02475	0.000117	-0.244983949	-0.0697009
		Bs_AF		0.02475	1.09E-12	1.06572815	1.24101123
		Ba_SC		0.02475	0.029496	0.00633673	0.18161981
		Ba_AF		0.02475	2.07E-10	0.268336758	0.44361984
		Dw_SC		0.02475	1.55E-10	-0.449315493	-0.2740324
		Dw_AF		0.02475	1.09E-12	0.6072281	0.78251118
		Fb_SC		0.02475	6.43E-06	-0.280581779	-0.1052987
		Fb_AF		0.02475	1.09E-12	0.945407484	1.12069057
		Cp_AF		0.02475	1.09E-12	0.974597705	1.14988079
	Cp_AF	Bs_SC		0.02475	1.09E-12	-1.307223195	-1.1319401
		Bs_AF		0.02475	0.037468	0.003488904	0.17877199
		Ba_SC		0.02475	1.09E-12	-1.055902516	-0.8806194
		Ba_AF		0.02475	1.09E-12	-0.793902487	-0.6186194
		Dw_SC		0.02475	1.09E-12	-1.511554739	-1.3362717
		Dw_AF		0.02475	1.17E-10	-0.455011146	-0.2797281
		Fb_SC		0.02475	1.09E-12	-1.342821025	-1.1675379
		Fb_AF	-0.029190221	0.02475	0.967501	-0.116831762	0.05845132
		Cp_SC		0.02475	1.09E-12	-1.149880787	-0.9745977
*. The mean difference is significant at the 0.05 level							

Supplementary Table S6. HSD-Tukey test for comparison of the relative abundance of rhizospheric fungal communities of cereals (barely and durum wheat) and legumes (chickpea and faba bean) crops in Agroforestry (AF) and sole cropping (SC) systems. Bs, bulk soil; Ba, barely; Dw, durum wheat; Cp, chickpeas; Fb, Faba bean

Dependent Variable	traitments		Mean Difference (I-J)	Std. Error	Sig.	95% Confidence Interval	
						Lower Bound	Upper Bound
**Chytridiomycetes**	Bs_FS	Bs_AF		0.00290073	1.273E-09	0.027461896	0.0480055
		Ba_FS		0.00290073	0.00031549	0.006815154	0.02735876
		Ba_AF		0.00290073	0.00031549	0.006815154	0.02735876
		Dw_FS	-0.002847826	0.00290073	0.99016258	-0.01311963	0.00742398
		Dw_AF		0.00290073	3.4303E-10	0.030309722	0.05085333
		Fb_FS	-0.003559783	0.00290073	0.95869524	-0.013831587	0.00671202
		Fb_AF	0.009255435	0.00290073	0.09963046	-0.001016368	0.01952724
		Cp_FS		0.00290073	1.0972E-07	0.018918417	0.03946202
		Cp_AF		0.00290073	2.2649E-06	0.01393472	0.03447833
	Bs_AF	Bs_FS		0.00290073	1.273E-09	-0.048005503	-0.0274619
		Ba_FS		0.00290073	2.4492E-05	-0.030918545	-0.0103749
		Ba_AF		0.00290073	2.4492E-05	-0.030918545	-0.0103749
		Dw_FS		0.00290073	3.4303E-10	-0.05085333	-0.0303097
		Dw_AF	0.002847826	0.00290073	0.99016258	-0.007423978	0.01311963
		Fb_FS		0.00290073	2.5018E-10	-0.051565287	-0.0310217
		Fb_AF		0.00290073	1.6558E-07	-0.038750068	-0.0182065
		Cp_FS	-0.008543479	0.00290073	0.15658789	-0.018815283	0.00172832
		Cp_AF		0.00290073	0.00456634	-0.023798979	-0.0032554
	Ba_FS	Bs_FS		0.00290073	0.00031549	-0.027358762	-0.0068152
		Bs_AF		0.00290073	2.4492E-05	0.010374938	0.03091855
		Ba_AF	0	0.00290073	1	-0.010271804	0.0102718
		Dw_FS		0.00290073	4.03E-05	-0.030206588	-0.009663
		Dw_AF		0.00290073	3.5927E-06	0.013222764	0.03376637
		Fb_FS		0.00290073	2.4492E-05	-0.030918545	-0.0103749
		Fb_AF	-0.007831523	0.00290073	0.23804404	-0.018103326	0.00244028
		Cp_FS		0.00290073	0.01328074	0.001831459	0.02237507
		Cp_AF	0.007119566	0.00290073	0.34723228	-0.003152238	0.01739137
	Ba_AF	Bs_FS		0.00290073	0.00031549	-0.027358762	-0.0068152
		Bs_AF		0.00290073	2.4492E-05	0.010374938	0.03091855
		Ba_FS	0	0.00290073	1	-0.010271804	0.0102718
		Dw_FS		0.00290073	4.03E-05	-0.030206588	-0.009663
		Dw_AF		0.00290073	3.5927E-06	0.013222764	0.03376637
		Fb_FS		0.00290073	2.4492E-05	-0.030918545	-0.0103749
		Fb_AF	-0.007831523	0.00290073	0.23804404	-0.018103326	0.00244028
		Cp_FS		0.00290073	0.01328074	0.001831459	0.02237507
		Cp_AF	0.007119566	0.00290073	0.34723228	-0.003152238	0.01739137

	Dw_FS	Bs_FS	0.002847826	0.00290073	0.99016258	-0.007423977	0.01311963
		Bs_AF		0.00290073	3.4303E-10	0.030309722	0.05085333
		Ba_FS		0.00290073	4.03E-05	0.009662981	0.03020659
		Ba_AF		0.00290073	4.03E-05	0.009662981	0.03020659
		Dw_AF	-1,04586*	0.04886429	1.1949E-12	-1.218897978	-0.8728305
		Fb_FS	-0.000711957	0.00290073	0.99999989	-0.010983761	0.00955985
		Fb_AF		0.00290073	0.01328075	0.001831458	0.02237507
		Cp_FS		0.00290073	2.2587E-08	0.021766243	0.04230985
		Cp_AF		0.00290073	3.8489E-07	0.016782547	0.03732615
	Dw_AF	Bs_FS		0.00290073	3.4303E-10	-0.050853329	-0.0303097
		Bs_AF	-0.002847826	0.00290073	0.99016258	-0.01311963	0.00742398
		Ba_FS		0.00290073	3.5927E-06	-0.033766371	-0.0132228
		Ba_AF		0.00290073	3.5927E-06	-0.033766371	-0.0132228
		Dw_FS	-1,04586*	0.04886429	1.1949E-12	-1.218897978	-0.8728305
		Fb_FS		0.00290073	7.4266E-11	-0.054413113	-0.0338695
		Fb_AF		0.00290073	3.3215E-08	-0.041597894	-0.0210543
		Cp_FS		0.00290073	0.02243842	-0.021663109	-0.0011195
		Cp_AF		0.00290073	0.00053519	-0.026646805	-0.0061032
	Fb_FS	Bs_FS	0.003559783	0.00290073	0.95869524	-0.00671202	0.01383159
		Bs_AF		0.00290073	2.5018E-10	0.031021679	0.05156529
		Ba_FS		0.00290073	2.4492E-05	0.010374938	0.03091855
		Ba_AF		0.00290073	2.4492E-05	0.010374938	0.03091855
		Dw_FS	0.000711957	0.00290073	0.99999989	-0.009559847	0.01098376
		Dw_AF		0.00290073	7.4266E-11	0.033869505	0.05441311
		Fb_AF		0.00290073	0.00780406	0.002543415	0.02308702
		Cp_FS		0.00290073	1.5454E-08	0.0224782	0.04302181
		Cp_AF		0.00290073	2.5158E-07	0.017494504	0.03803811
	Fb_AF	Bs_FS	-0.009255435	0.00290073	0.09963046	-0.019527239	0.00101637
		Bs_AF		0.00290073	1.6558E-07	0.018206461	0.03875007
		Ba_FS	0.007831523	0.00290073	0.23804404	-0.002440281	0.01810333
		Ba_AF	0.007831523	0.00290073	0.23804404	-0.002440281	0.01810333
		Dw_FS		0.00290073	0.01328075	-0.022375065	-0.0018315
		Dw_AF		0.00290073	3.3215E-08	0.021054287	0.04159789
		Fb_FS		0.00290073	0.00780406	-0.023087022	-0.0025434
		Cp_FS		0.00290073	4.03E-05	0.009662981	0.03020659
		Cp_AF		0.00290073	0.00155793	0.004679285	0.02522289
	Cp_FS	Bs_FS		0.00290073	1.0972E-07	-0.039462024	-0.0189184
		Bs_AF	0.008543479	0.00290073	0.15658789	-0.001728324	0.01881528
		Ba_FS		0.00290073	0.01328074	-0.022375066	-0.0018315
		Ba_AF		0.00290073	0.01328074	-0.022375066	-0.0018315
		Dw_FS		0.00290073	2.2587E-08	-0.04230985	-0.0217662
		Dw_AF		0.00290073	0.02243842	0.001119502	0.02166311
		Fb_FS		0.00290073	1.5454E-08	-0.043021807	-0.0224782
		Fb_AF		0.00290073	4.03E-05	-0.030206589	-0.009663

		Cp_AF	-0.004983696	0.00290073	0.77372559	-0.0152555	0.00528811
	Cp_AF	Bs_FS		0.00290073	2.2649E-06	-0.034478328	-0.0139347
		Bs_AF		0.00290073	0.00456634	0.003255372	0.02379898
		Ba_FS	-0.007119566	0.00290073	0.34723228	-0.01739137	0.00315224
		Ba_AF	-0.007119566	0.00290073	0.34723228	-0.01739137	0.00315224
		Dw_FS		0.00290073	3.8489E-07	-0.037326154	-0.0167825
		Dw_AF		0.00290073	0.00053519	0.006103198	0.02664681
		Fb_FS		0.00290073	2.5158E-07	-0.038038111	-0.0174945
		Fb_AF		0.00290073	0.00155793	-0.025222892	-0.0046793
		Cp_FS	0.004983696	0.00290073	0.77372559	-0.005288107	0.0152555
**Sordariomycetes**	Bs_FS	Bs_AF	,85008*	0.04434257	1.9734E-12	0.693054354	1.007098
		Ba_FS	-,21928*	0.04434257	0.00246256	-0.376304458	-0.0622608
		Ba_AF	,53041*	0.04434257	5.6405E-09	0.373385841	0.68742949
		Dw_FS	0.083298922	0.04434257	0.68213285	-0.073722903	0.24032075
		Dw_AF	,58238*	0.04434257	1.072E-09	0.425358673	0.73940232
		Fb_FS	0.004271739	0.04434257	1	-0.152750086	0.16129356
		Fb_AF	,71765*	0.04434257	2.4364E-11	0.560630427	0.87467408
		Cp_FS	,22996*	0.04434257	0.00145331	0.072940157	0.38698381
		Cp_AF	1,01383*	0.04434257	1.1093E-12	0.856804372	1.17084802
	Bs_AF	Bs_FS	-,85008*	0.04434257	1.9734E-12	-1.007098004	-0.6930544
		Ba_FS	-1,06936*	0.04434257	1.0962E-12	-1.226380637	-0.912337
		Ba_AF	-,31967*	0.04434257	2.0387E-05	-0.476690338	-0.1626467
		Dw_FS	-,76678*	0.04434257	7.7045E-12	-0.923799082	-0.6097554
		Dw_AF	-,26770*	0.04434257	0.00023071	-0.424717506	-0.1106739
		Fb_FS	-,84580*	0.04434257	2.0658E-12	-1.002826265	-0.6887826
		Fb_AF	-0.132423927	0.04434257	0.14547766	-0.289445752	0.0245979
		Cp_FS	-,62011*	0.04434257	3.4547E-10	-0.777136023	-0.4630924
		Cp_AF	,16375*	0.04434257	0.03664341	0.006728193	0.32077184
	Ba_FS	Bs_FS	,21928*	0.04434257	0.00246256	0.062260808	0.37630446
		Bs_AF	1,06936*	0.04434257	1.0962E-12	0.912336987	1.22638064
		Ba_AF	,74969*	0.04434257	1.1284E-11	0.592668474	0.90671212
		Dw_FS	,30258*	0.04434257	4.4514E-05	0.14555973	0.45960338
		Dw_AF	,80166*	0.04434257	3.8913E-12	0.644641306	0.95868496
		Fb_FS	,22355*	0.04434257	0.00199395	0.066532547	0.3805762
		Fb_AF	,93693*	0.04434257	1.2219E-12	0.77991306	1.09395671
		Cp_FS	,44924*	0.04434257	9.7991E-08	0.292222789	0.60626644
		Cp_AF	1,23311*	0.04434257	1.0917E-12	1.076087005	1.39013066
	Ba_AF	Bs_FS	-,53041*	0.04434257	5.6405E-09	-0.687429491	-0.3733858
		Bs_AF	,31967*	0.04434257	2.0387E-05	0.162646688	0.47669034
		Ba_FS	-,74969*	0.04434257	1.1284E-11	-0.906712124	-0.5926685
		Dw_FS	-,44711*	0.04434257	1.0614E-07	-0.604130569	-0.2900869
		Dw_AF	0.051972832	0.04434257	0.96872338	-0.105048993	0.20899466
		Fb_FS	-,52614*	0.04434257	6.501E-09	-0.683157752	-0.3691141
		Fb_AF	,18724*	0.04434257	0.01191815	0.030222761	0.34426641

		Cp_FS	-,30045*	0.04434257	4.9137E-05	-0.45746751	-0.1434239
		Cp_AF	,48342*	0.04434257	2.8252E-08	0.326396706	0.64044036
	Dw_FS	Bs_FS	-0.083298922	0.04434257	0.68213285	-0.240320747	0.0737229
		Bs_AF	,76678*	0.04434257	7.7045E-12	0.609755432	0.92379908
		Ba_FS	-,30258*	0.04434257	4.4514E-05	-0.45960338	-0.1455597
		Ba_AF	,44711*	0.04434257	1.0614E-07	0.290086919	0.60413057
		Dw_AF	,49908*	0.04434257	1.631E-08	0.342059751	0.6561034
		Fb_FS	-0.079027183	0.04434257	0.73828714	-0.236049008	0.07799464
		Fb_AF	,63435*	0.04434257	2.2878E-10	0.477331505	0.79137515
		Cp_FS	0.146663059	0.04434257	0.07957743	-0.010358766	0.30368488
		Cp_AF	,93053*	0.04434257	1.2399E-12	0.77350545	1.0875491
	Dw_AF	Bs_FS	-,58238*	0.04434257	1.072E-09	-0.739402323	-0.4253587
		Bs_AF	,26770*	0.04434257	0.00023071	0.110673856	0.42471751
		Ba_FS	-,80166*	0.04434257	3.8913E-12	-0.958684956	-0.6446413
		Ba_AF	-0.051972832	0.04434257	0.96872338	-0.208994657	0.10504899
		Dw_FS	-,49908*	0.04434257	1.631E-08	-0.656103401	-0.3420598
		Fb_FS	-,57811*	0.04434257	1.2232E-09	-0.735130584	-0.4210869
		Fb_AF	0.135271754	0.04434257	0.12944266	-0.021750071	0.29229358
		Cp_FS	-,35242*	0.04434257	4.7978E-06	-0.509440341	-0.1953967
		Cp_AF	,43145*	0.04434257	1.9215E-07	0.274423874	0.58846752
	Fb_FS	Bs_FS	-0.004271739	0.04434257	1	-0.161293564	0.15275009
		Bs_AF	,84580*	0.04434257	2.0658E-12	0.688782615	1.00282627
		Ba_FS	-,22355*	0.04434257	0.00199395	-0.380576197	-0.0665325
		Ba_AF	,52614*	0.04434257	6.501E-09	0.369114102	0.68315775
		Dw_FS	0.079027183	0.04434257	0.73828714	-0.077994642	0.23604901
		Dw_AF	,57811*	0.04434257	1.2232E-09	0.421086934	0.73513058
		Fb_AF	,71338*	0.04434257	2.711E-11	0.556358688	0.87040234
		Cp_FS	,22569*	0.04434257	0.00179433	0.068668417	0.38271207
		Cp_AF	1,00955*	0.04434257	1.1111E-12	0.852532633	1.16657628
	Fb_AF	Bs_FS	-,71765*	0.04434257	2.4364E-11	-0.874674077	-0.5606304
		Bs_AF	0.132423927	0.04434257	0.14547766	-0.024597898	0.28944575
		Ba_FS	-,93693*	0.04434257	1.2219E-12	-1.09395671	-0.7799131
		Ba_AF	-,18724*	0.04434257	0.01191815	-0.344266411	-0.0302228
		Dw_FS	-,63435*	0.04434257	2.2878E-10	-0.791375155	-0.4773315
		Dw_AF	-0.135271754	0.04434257	0.12944266	-0.292293579	0.02175007
		Fb_FS	-,71338*	0.04434257	2.711E-11	-0.870402338	-0.5563587
		Cp_FS	-,48769*	0.04434257	2.429E-08	-0.644712095	-0.3306684
		Cp_AF	,29617*	0.04434257	5.9922E-05	0.13915212	0.45319577
	Cp_FS	Bs_FS	-,22996*	0.04434257	0.00145331	-0.386983807	-0.0729402
		Bs_AF	,62011*	0.04434257	3.4547E-10	0.463092373	0.77713602
		Ba_FS	-,44924*	0.04434257	9.7991E-08	-0.606266439	-0.2922228
		Ba_AF	,30045*	0.04434257	4.9137E-05	0.14342386	0.45746751
		Dw_FS	-0.146663059	0.04434257	0.07957743	-0.303684884	0.01035877
		Dw_AF	,35242*	0.04434257	4.7978E-06	0.195396691	0.50944034

		Fb_FS	-,22569*	0.04434257	0.00179433	-0.382712067	-0.0686684
		Fb_AF	,48769*	0.04434257	2.429E-08	0.330668445	0.6447121
		Cp_AF	,78386*	0.04434257	5.4199E-12	0.626842391	0.94088604
	Cp_AF	Bs_FS	-1,01383*	0.04434257	1.1093E-12	-1.170848022	-0.8568044
		Bs_AF	-,16375*	0.04434257	0.03664341	-0.320771843	-0.0067282
		Ba_FS	-1,23311*	0.04434257	1.0917E-12	-1.390130655	-1.076087
		Ba_AF	-,48342*	0.04434257	2.8252E-08	-0.640440356	-0.3263967
		Dw_FS	-,93053*	0.04434257	1.2399E-12	-1.0875491	-0.7735054
		Dw_AF	-,43145*	0.04434257	1.9215E-07	-0.588467524	-0.2744239
		Fb_FS	-1,00955*	0.04434257	1.1111E-12	-1.166576283	-0.8525326
		Fb_AF	-,29617*	0.04434257	5.9922E-05	-0.45319577	-0.1391521
		Cp_FS	-,78386*	0.04434257	5.4199E-12	-0.940886041	-0.6268424
**Dothideomycetes**	Bs_FS	Bs_AF	0.012103262	0.00593107	0.58348456	-0.008899306	0.03310583
		Ba_FS	0.008543479	0.00593107	0.89961735	-0.012459089	0.02954605
		Ba_AF	0.014239132	0.00593107	0.37471051	-0.006763437	0.0352417
		Dw_FS		0.00593107	0.00741484	-0.047344963	-0.0053398
		Dw_AF	0.002847827	0.00593107	0.99996276	-0.018154742	0.0238504
		Fb_FS		0.00593107	1.0916E-12	-0.333551515	-0.2915464
		Fb_AF	0.020646741	0.00593107	0.05643522	-0.000355827	0.04164931
		Cp_FS	0.00213587	0.00593107	0.99999682	-0.018866699	0.02313844
		Cp_AF	-3.33333E-10	0.00593107	1	-0.021002569	0.02100257
	Bs_AF	Bs_FS	-0.012103262	0.00593107	0.58348456	-0.03310583	0.00889931
		Ba_FS	-0.003559783	0.00593107	0.9997636	-0.024562351	0.01744279
		Ba_AF	0.00213587	0.00593107	0.99999682	-0.018866699	0.02313844
		Dw_FS		0.00593107	9.0228E-05	-0.059448225	-0.0174431
		Dw_AF	-0.009255435	0.00593107	0.85148177	-0.030258004	0.01174713
		Fb_FS		0.00593107	1.0916E-12	-0.345654777	-0.3036496
		Fb_AF	0.008543479	0.00593107	0.89961733	-0.012459089	0.02954605
		Cp_FS	-0.009967392	0.00593107	0.79356215	-0.030969961	0.01103518
		Cp_AF	-0.012103262	0.00593107	0.58348452	-0.033105831	0.00889931
	Ba_FS	Bs_FS	-0.008543479	0.00593107	0.89961735	-0.029546047	0.01245909
		Bs_AF	0.003559783	0.00593107	0.9997636	-0.017442785	0.02456235
		Ba_AF	0.005695653	0.00593107	0.99155593	-0.015306916	0.02669822
		Dw_FS		0.00593107	0.00032142	-0.055888442	-0.0138833
		Dw_AF	-0.005695652	0.00593107	0.99155593	-0.026698221	0.01530692
		Fb_FS		0.00593107	1.0916E-12	-0.342094994	-0.3000899
		Fb_AF	0.012103262	0.00593107	0.58348452	-0.008899306	0.03310583
		Cp_FS	-0.006407609	0.00593107	0.98133447	-0.027410178	0.01459496
		Cp_AF	-0.008543479	0.00593107	0.89961733	-0.029546048	0.01245909
	Ba_AF	Bs_FS	-0.014239132	0.00593107	0.37471051	-0.0352417	0.00676344
		Bs_AF	-0.00213587	0.00593107	0.99999682	-0.023138438	0.0188667
		Ba_FS	-0.005695653	0.00593107	0.99155593	-0.026698221	0.01530692
		Dw_FS		0.00593107	4.2862E-05	-0.061584094	-0.019579
		Dw_AF	-0.011391305	0.00593107	0.65683189	-0.032393873	0.00961126

		Fb_FS		0.00593107	1.0916E-12	-0.347790647	-0.3057855
		Fb_AF	0.00640761	0.00593107	0.98133446	-0.014594959	0.02741018
		Cp_FS	-0.012103262	0.00593107	0.58348456	-0.03310583	0.00889931
		Cp_AF	-0.014239132	0.00593107	0.37471048	-0.0352417	0.00676344
	Dw_FS	Bs_FS		0.00593107	0.00741484	0.005339826	0.04734496
		Bs_AF		0.00593107	9.0228E-05	0.017443088	0.05944822
		Ba_FS		0.00593107	0.00032142	0.013883305	0.05588844
		Ba_AF		0.00593107	4.2862E-05	0.019578958	0.06158409
		Dw_AF		0.00593107	0.0025934	0.008187653	0.05019279
		Fb_FS		0.00593107	1.0916E-12	-0.307209121	-0.265204
		Fb_AF		0.00593107	5.0341E-06	0.025986567	0.0679917
		Cp_FS		0.00593107	0.00337391	0.007475696	0.04948083
		Cp_AF		0.00593107	0.00741484	0.005339826	0.04734496
	Dw_AF	Bs_FS	-0.002847827	0.00593107	0.99996276	-0.023850395	0.01815474
		Bs_AF	0.009255435	0.00593107	0.85148177	-0.011747133	0.030258
		Ba_FS	0.005695652	0.00593107	0.99155593	-0.015306916	0.02669822
		Ba_AF	0.011391305	0.00593107	0.65683189	-0.009611263	0.03239387
		Dw_FS		0.00593107	0.0025934	-0.050192789	-0.0081877
		Fb_FS		0.00593107	1.0916E-12	-0.336399342	-0.2943942
		Fb_AF	0.017798915	0.00593107	0.14169875	-0.003203654	0.03880148
		Cp_FS	-0.000711957	0.00593107	1	-0.021714525	0.02029061
		Cp_AF	-0.002847827	0.00593107	0.99996276	-0.023850395	0.01815474
	Fb_FS	Bs_FS		0.00593107	1.0916E-12	0.291546379	0.33355152
		Bs_AF		0.00593107	1.0916E-12	0.303649641	0.34565478
		Ba_FS		0.00593107	1.0916E-12	0.300089858	0.34209499
		Ba_AF		0.00593107	1.0916E-12	0.30578551	0.34779065
		Dw_FS		0.00593107	1.0916E-12	0.265203984	0.30720912
		Dw_AF		0.00593107	1.0916E-12	0.294394205	0.33639934
		Fb_AF	-,33960*	0.00749868	1.0916E-12	-0.366156916	-0.3130497
		Cp_FS		0.00593107	1.0916E-12	0.293682248	0.33568739
		Cp_AF		0.00593107	1.0916E-12	0.291546378	0.33355152
	Fb_AF	Bs_FS	-0.020646741	0.00593107	0.05643522	-0.04164931	0.00035583
		Bs_AF	-0.008543479	0.00593107	0.89961733	-0.029546048	0.01245909
		Ba_FS	-0.012103262	0.00593107	0.58348452	-0.033105831	0.00889931
		Ba_AF	-0.00640761	0.00593107	0.98133446	-0.027410178	0.01459496
		Dw_FS		0.00593107	5.0341E-06	-0.067991704	-0.0259866
		Dw_AF	-0.017798915	0.00593107	0.14169875	-0.038801483	0.00320365
		Fb_FS	-,33960*	0.00749868	1.0916E-12	-0.366156916	-0.3130497
		Cp_FS	-0.018510872	0.00593107	0.11362173	-0.03951344	0.0024917
		Cp_AF	-0.020646742	0.00593107	0.05643522	-0.04164931	0.00035583
	Cp_FS	Bs_FS	-0.00213587	0.00593107	0.99999682	-0.023138438	0.0188667
		Bs_AF	0.009967392	0.00593107	0.79356215	-0.011035176	0.03096996
		Ba_FS	0.006407609	0.00593107	0.98133447	-0.014594959	0.02741018
		Ba_AF	0.012103262	0.00593107	0.58348456	-0.008899306	0.03310583

		Dw_FS		0.00593107	0.00337391	-0.049480832	-0.0074757
		Dw_AF	0.000711957	0.00593107	1	-0.020290611	0.02171453
		Fb_FS		0.00593107	1.0916E-12	-0.335687385	-0.2936822
		Fb_AF	0.018510872	0.00593107	0.11362173	-0.002491697	0.03951344
		Cp_AF	-0.00213587	0.00593107	0.99999682	-0.023138438	0.0188667
	Cp_AF	Bs_FS	3.33333E-10	0.00593107	1	-0.021002568	0.02100257
		Bs_AF	0.012103262	0.00593107	0.58348452	-0.008899306	0.03310583
		Ba_FS	0.008543479	0.00593107	0.89961733	-0.012459089	0.02954605
		Ba_AF	0.014239132	0.00593107	0.37471048	-0.006763436	0.0352417
		Dw_FS		0.00593107	0.00741484	-0.047344962	-0.0053398
		Dw_AF	0.002847827	0.00593107	0.99996276	-0.018154741	0.0238504
		Fb_FS		0.00593107	1.0916E-12	-0.333551515	-0.2915464
		Fb_AF	0.020646742	0.00593107	0.05643522	-0.000355827	0.04164931
		Cp_FS	0.00213587	0.00593107	0.99999682	-0.018866698	0.02313844
**Lecanoromycetes**	Bs_FS	Bs_AF	0.021358698	0.03600793	0.99978518	-0.106149256	0.14886665
		Ba_FS		0.03600793	0.00031444	-0.33967102	-0.0846551
		Ba_AF		0.03600793	9.6077E-08	-0.492741689	-0.2377258
		Dw_FS		0.03600793	0.00073832	-0.325431888	-0.070416
		Dw_AF		0.03600793	0.00281765	0.048345326	0.30336123
		Fb_FS	0.008543479	0.00992322	0.55900406	-0.055785937	0.01449245
		Fb_AF		0.03600793	4.1247E-05	0.119540986	0.37455689
		Cp_FS		0.03600793	5.8482E-10	-0.616622138	-0.3616062
		Cp_AF		0.03600793	7.9207E-05	-0.363165588	-0.1081497
	Bs_AF	Bs_FS	-0.021358698	0.03600793	0.99978518	-0.148866652	0.10614926
		Ba_FS		0.03600793	8.9625E-05	-0.361029718	-0.1060138
		Ba_AF		0.03600793	3.6711E-08	-0.514100387	-0.2590845
		Dw_FS	0.008543479	0.00992322	0.55900406	-0.055785937	0.01449245
		Dw_AF		0.03600793	0.01029053	0.026986628	0.28200254
		Fb_FS		0.03600793	6.9126E-09	0.298242092	0.553258
		Fb_AF	0.008543479	0.00992322	0.55900406	-0.055785937	0.01449245
		Cp_FS		0.03600793	2.6973E-10	-0.637980836	-0.3829649
		Cp_AF		0.03600793	2.3525E-05	-0.384524286	-0.1295084
	Ba_FS	Bs_FS		0.03600793	0.00031444	0.084655112	0.33967102
		Bs_AF		0.03600793	8.9625E-05	0.10601381	0.36102972
		Ba_AF	,22569*	0.03600793	0.00014144	0.098182288	0.3531982
		Dw_FS	0.014239132	0.03600793	0.99999287	-0.113268822	0.14174709
		Dw_AF		0.03600793	3.4476E-08	0.260508392	0.5155243
		Fb_FS		0.03600793	3.2778E-12	0.531763857	0.78677976
		Fb_AF		0.03600793	1.8139E-09	0.331704052	0.58671996
		Cp_FS		0.03600793	7.8659E-06	-0.404459071	-0.1494432
		Cp_AF	-0.023494568	0.03600793	0.99953696	-0.151002522	0.10401339
	Ba_AF	Bs_FS		0.03600793	9.6077E-08	0.237725781	0.49274169
		Bs_AF		0.03600793	3.6711E-08	0.259084479	0.51410039
		Ba_FS	,22569*	0.03600793	0.00014144	0.098182288	0.3531982

		Dw_FS		0.03600793	0.00473835	0.039801847	0.29481775
		Dw_AF		0.03600793	9.3419E-11	0.413579061	0.66859497
		Fb_FS		0.03600793	1.1161E-12	0.684834525	0.93985043
		Fb_AF		0.03600793	1.0233E-11	0.484774721	0.73979063
		Cp_FS	-0.123880449	0.03600793	0.06123255	-0.251388403	0.00362751
		Cp_AF		0.03600793	0.04448337	0.002068147	0.25708406
	Dw_FS	Bs_FS		0.03600793	0.00073832	0.07041598	0.32543189
		Bs_AF		0.03600793	0.00020616	0.091774678	0.34679059
		Ba_FS	-0.014239132	0.03600793	0.99999287	-0.141747086	0.11326882
		Ba_AF		0.03600793	0.00473835	-0.294817755	-0.0398018
		Dw_AF		0.03600793	6.5091E-08	0.24626926	0.50128517
		Fb_FS		0.03600793	4.4387E-12	0.517524725	0.77254063
		Fb_AF		0.03600793	3.1748E-09	0.31746492	0.57248083
		Cp_FS		0.03600793	3.6802E-06	0.869820346	-0.1636823
		Cp_AF	-0.0377337	0.03600793	0.98471929	-0.165241654	0.08977425
	Dw_AF	Bs_FS		0.03600793	0.00281765	-0.303361234	-0.0483453
		Bs_AF		0.03600793	0.01029053	-0.282002536	-0.0269866
		Ba_FS		0.03600793	3.4476E-08	-0.5155243	-0.2605084
		Ba_AF		0.03600793	9.3419E-11	-0.668594969	-0.4135791
		Dw_FS		0.03600793	6.5091E-08	-0.501285168	-0.2462693
		Fb_FS		0.03600793	1.0716E-05	0.14374751	0.39876342
		Fb_AF	0.07119566	0.03600793	0.62236372	-0.056312294	0.19870361
		Cp_FS		0.03600793	2.9384E-12	-0.792475418	-0.5374595
		Cp_AF		0.03600793	1.2516E-08	0.539018868	-0.284003
	Fb_FS	Bs_FS		0.03600793	2.9166E-09	0.574616698	-0.3196008
		Bs_AF		0.03600793	6.9126E-09	-0.553258	-0.2982421
		Ba_FS		0.03600793	3.2778E-12	-0.786779765	-0.5317639
		Ba_AF		0.03600793	1.1161E-12	-0.939850433	-0.6848345
		Dw_FS		0.03600793	4.4387E-12	-0.772540633	-0.5175247
		Dw_AF		0.03600793	1.0716E-05	-0.398763418	-0.1437475
		Fb_AF		0.03600793	0.00064914	-0.327567758	-0.0725519
		Cp_FS		0.03600793	1.092E-12	-1.063730882	-0.808715
		Cp_AF		0.03600793	2.1878E-12	-0.810274332	-0.5552584
	Fb_AF	Bs_FS		0.03600793	4.1247E-05	-0.374556894	-0.119541
		Bs_AF		0.03600793	0.91414388	-0.353198196	-0.0981823
		Ba_FS		0.03600793	1.8139E-09	-0.58671996	-0.3317041
		Ba_AF		0.03600793	1.0233E-11	-0.739790629	-0.4847747
		Dw_FS		0.03600793	3.1748E-09	-0.572480828	-0.3174649
		Dw_AF	-0.07119566	0.03600793	0.62236372	-0.198703614	0.05631229
		Fb_FS		0.03600793	0.00064914	0.07255185	0.32756776
		Cp_FS		0.03600793	1.3367E-12	-0.863671078	-0.6086552
		Cp_AF		0.03600793	7.4174E-10	-0.610214528	-0.3551986
	Cp_FS	Bs_FS		0.03600793	5.8482E-10	0.36160623	0.61662214
		Bs_AF		0.03600793	2.6973E-10	0.382964928	0.63798084

		Ba_FS		0.03600793	7.8659E-06	0.149443163	0.40445907
		Ba_AF	0.123880449	0.03600793	0.06123255	-0.003627505	0.2513884
		Dw_FS		0.03600793	3.6802E-06	0.163682295	0.4186982
		Dw_AF		0.03600793	2.9384E-12	0.53745951	0.79247542
		Fb_FS		0.03600793	1.092E-12	0.808714974	1.06373088
		Fb_AF		0.03600793	1.3367E-12	0.60865517	0.86367108
		Cp_AF		0.03600793	2.872E-05	0.125948596	0.3809645
	Cp_AF	Bs_FS		0.03600793	7.9207E-05	0.10814968	0.36316559
		Bs_AF		0.03600793	2.3525E-05	0.129508378	0.38452429
		Ba_FS	0.023494568	0.03600793	0.99953696	-0.104013386	0.15100252
		Ba_AF		0.03600793	0.04448337	-0.257084055	-0.0020681
		Dw_FS	0.0377337	0.03600793	0.98471929	-0.089774254	0.16524165
		Dw_AF	,41151*	0.03600793	1.2516E-08	0.28400296	0.53901887
		Fb_FS		0.03600793	2.1878E-12	0.555258424	0.81027433
		Fb_AF		0.03600793	7.4174E-10	0.35519862	0.61021453
		Cp_FS		0.03600793	2.872E-05	-0.380964504	-0.1259486

*. The mean difference is significant at the 0.05 level

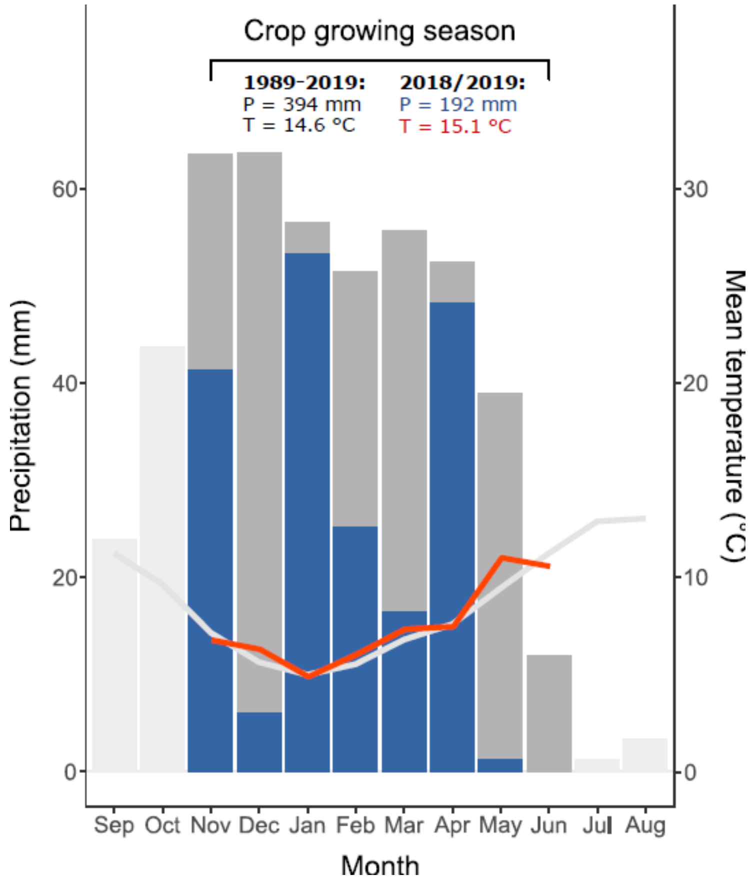

Supplementary Figure S1. The total monthly cumulative effective precipitation and temperature over the past 30 years in relation to 2018-2019. The means of temperature are indicated in grey for (1989-2019) and in red for (2018-2019). The histograms represent the mean of precipitation during 1989-2019 (grey) and 2018-2019 (blue).

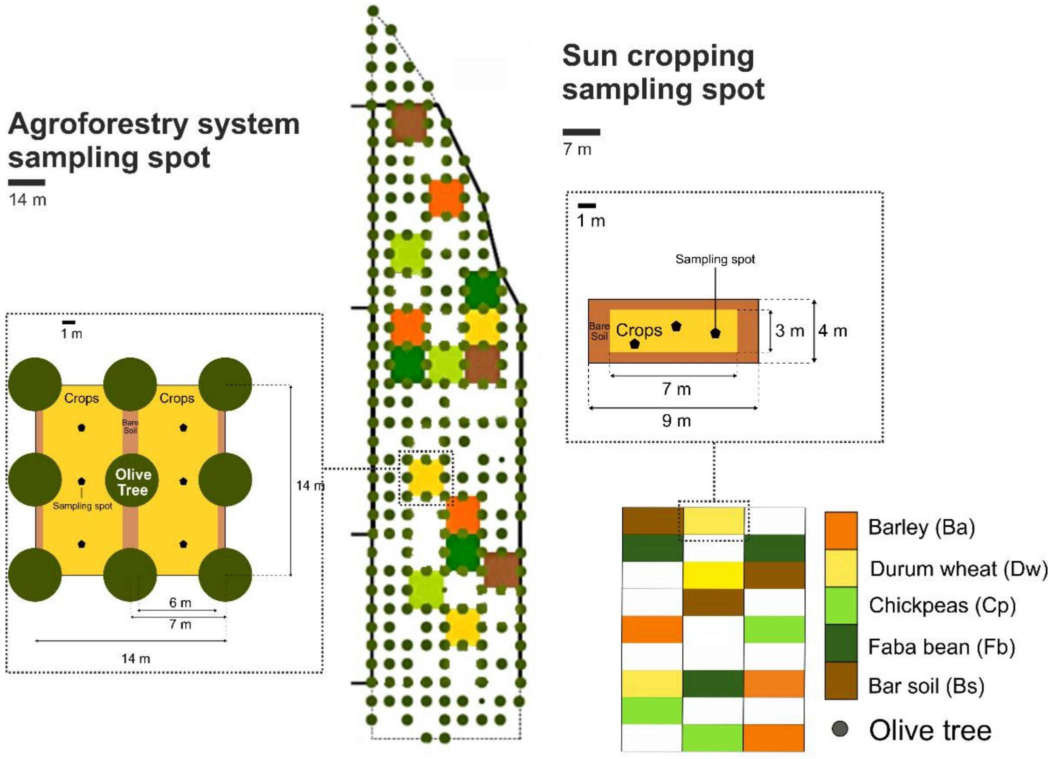

Fig.S2

Supplementary Figure S2. Experimental layout of agroforestry and sole cropping plots.

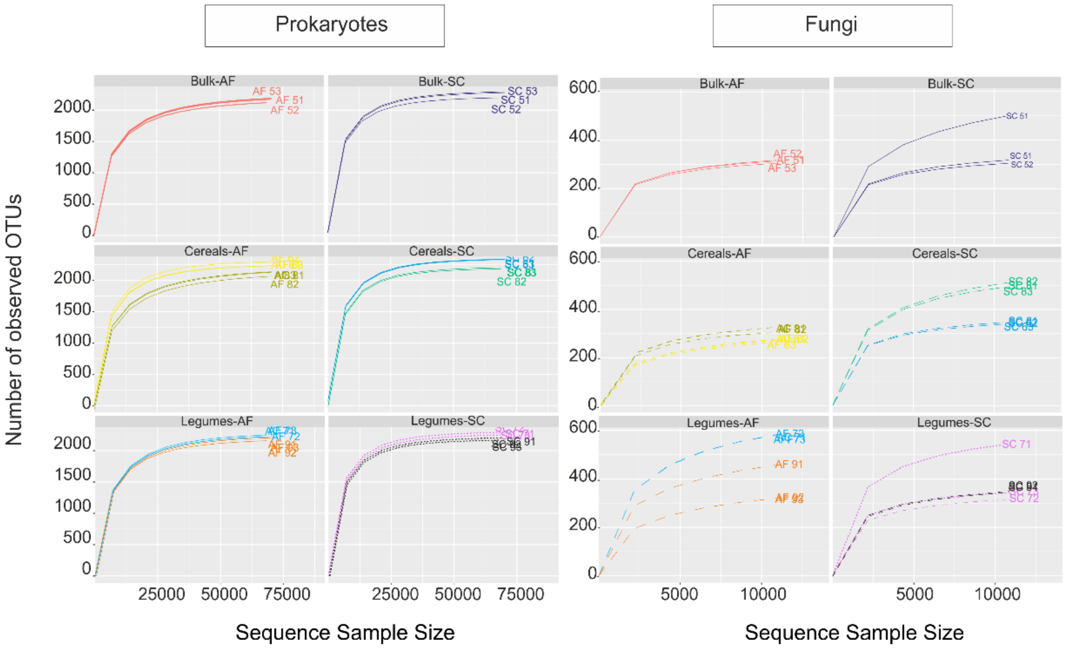

	Sample_tag#	Sample_name	Good's coverage %_Prokaryotes	Good's coverage %_Fungi
Agroforestry system	AF 51	Bulk_soil	99.8054750951326	99.6047794117647
	AF 52	Bulk_soil	99.7983756460499	99.5588235294118
	AF 53	Bulk_soil	99.8019253705912	99.5818014705882
	AF 61	Cereals_Barely	99.8835690350429	99.4852941176471
	AF 62	Cereals_Barely	99.8991878230249	99.4669117647059
	AF 63	Cereals_Barely	99.8913784290339	99.4761029411765
	AF 71	Legumes_Chickpeas	99.8480717896291	98.9797794117647
	AF 72	Legumes_Chickpeas	99.8636905776112	98.8877654196158
	AF 73	Legumes_Chickpeas	99.8558811836201	98.9337724156902
	AF 81	Cereals_Durum Wheat	99.788436417334	99.4944852941177
	AF 82	Cereals_Durum Wheat	99.7657181802692	99.6231617647059
	AF 83	Cereals_Durum Wheat	99.7770772988016	99.5588235294118
	AF 91	Legumes_Faba bean	99.8509115692622	99.0684375576462
	AF 92	Legumes_Faba bean	99.8764695859601	99.3474264705882
	AF 93	Legumes_Faba bean	99.8636905776112	99.2079320141172
Sun cropping	SC51	Bulk_soil	99.8977679332084	98.8371011256861
	SC52	Bulk_soil	99.9133867211904	99.6231617647059
	SC53	Bulk_soil	99.9055773271994	99.230131445196
	SC61	Cereals_Barely	99.9162265008235	99.5680147058824
	SC62	Cereals_Barely	99.9290055091725	99.6231617647059
	SC63	Cereals_Barely	99.922616004998	99.5955882352941
	SC71	Legumes_Chickpeas	99.9062872721077	99.2036841600307
	SC72	Legumes_Chickpeas	99.9176463906401	99.5955882352941
	SC73	Legumes_Chickpeas	99.9119668313739	99.3996361976624
	SC81	Cereals_Durum Wheat	99.9162265008235	99.1636029411765
	SC82	Cereals_Durum Wheat	99.9233259499063	99.0533088235294
	SC83	Cereals_Durum Wheat	99.9197762253649	99.108455882353
	SC91	Legumes_Faba bean	99.9091270517408	99.6231617647059
	SC92	Legumes_Faba bean	99.9176463906401	99.5036764705882
	SC93	Legumes_Faba bean	99.9133867211904	99.563419117647

Supplementary Figure S3. Rarefaction curves and Good's coverage percentage. To evaluate the sampling depth, rarefaction curves of the microbial communities of each crop rhizosphere sample in the agroforestry system (AF) and sun cropping (SC) systems based on 16 rDNA and ITS rDNA gene sequences.

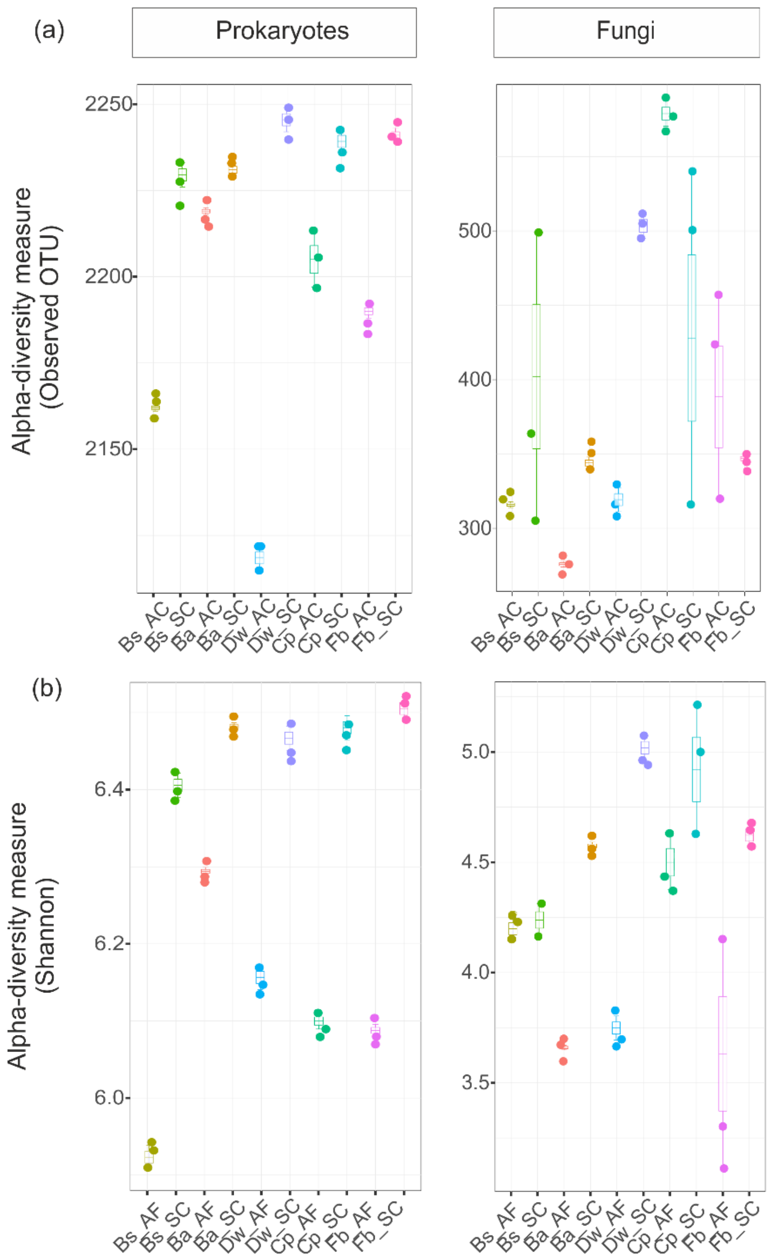

Supplementary Figure S4. Alpha diversity of cereals and legumes rhizospheric soil in the agroforestry system (AF) and sun cropping (SC) systems. (a) alpha diversity richness (observed OTU) for procaryotic (p-value: 0.02938; [Kruskal-Wallis] statistic: 18.542) and fungal (p-value: 0.12529; [Kruskal-Wallis] statistic: 13.918) communities. (b) alpha diversity Shannon indices for prokaryotic (Shannon: p-value: 0.027208; [Kruskal-Wallis] statistic: 18.771) and fungal (Shannon: p-value: 0.035506; [Kruskal-Wallis] statistic: 17.971) communities. Bs, bulk soil; Ba, barely; Dw, durum wheat; Cp, chickpeas; Fb, faba bean.

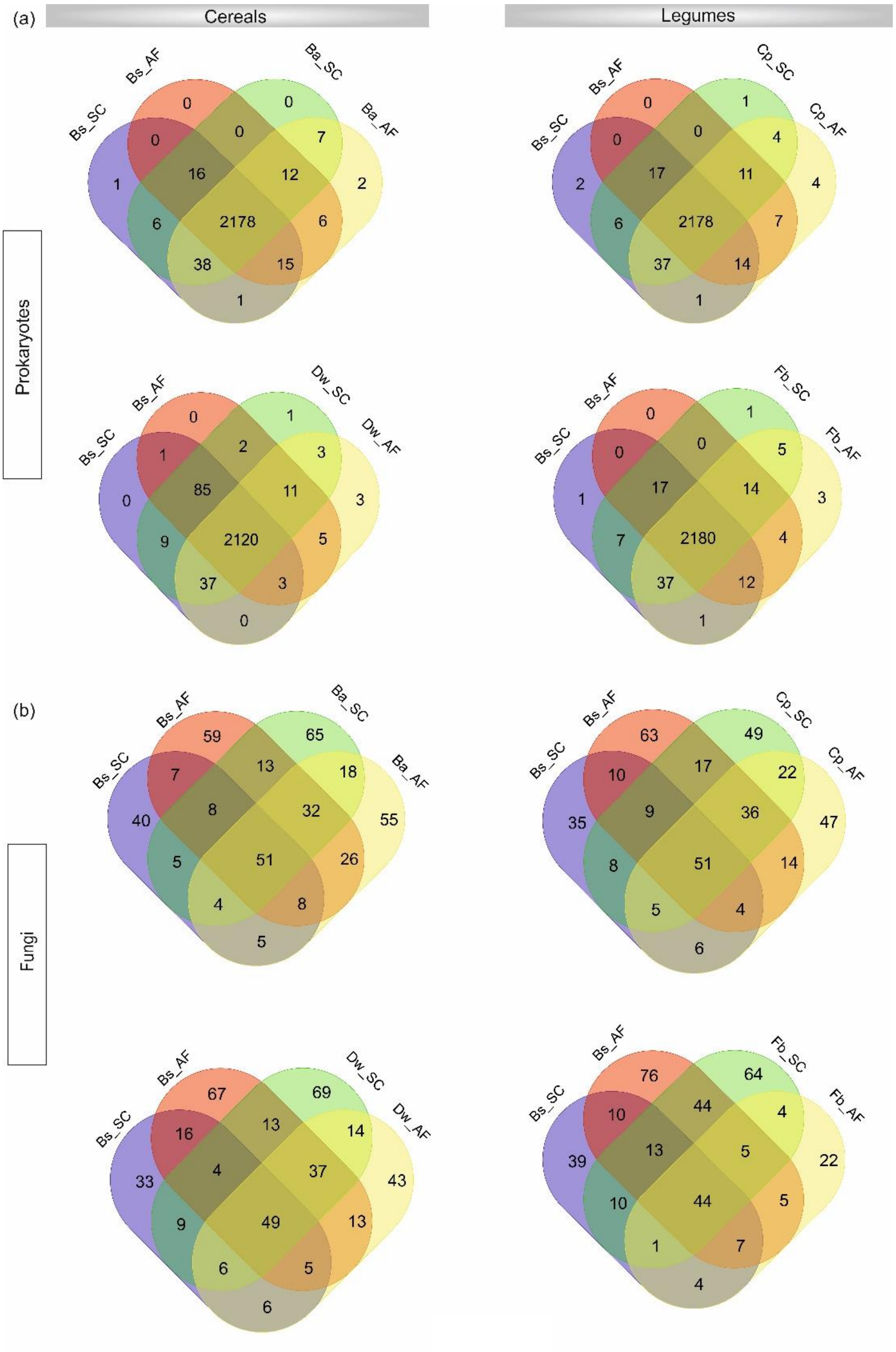

Supplementary Figure S5. Venn diagrams showing the distribution of prokaryotic (a) and fungal (b) measurable OTUs associated with cereals and legumes rhizospheric soil in the agroforestry system (AC) and sun cropping (SC) systems. Bs, bulk soil; Ba, barely; Dw, durum wheat; Cp, chickpeas; Fb, faba bean.

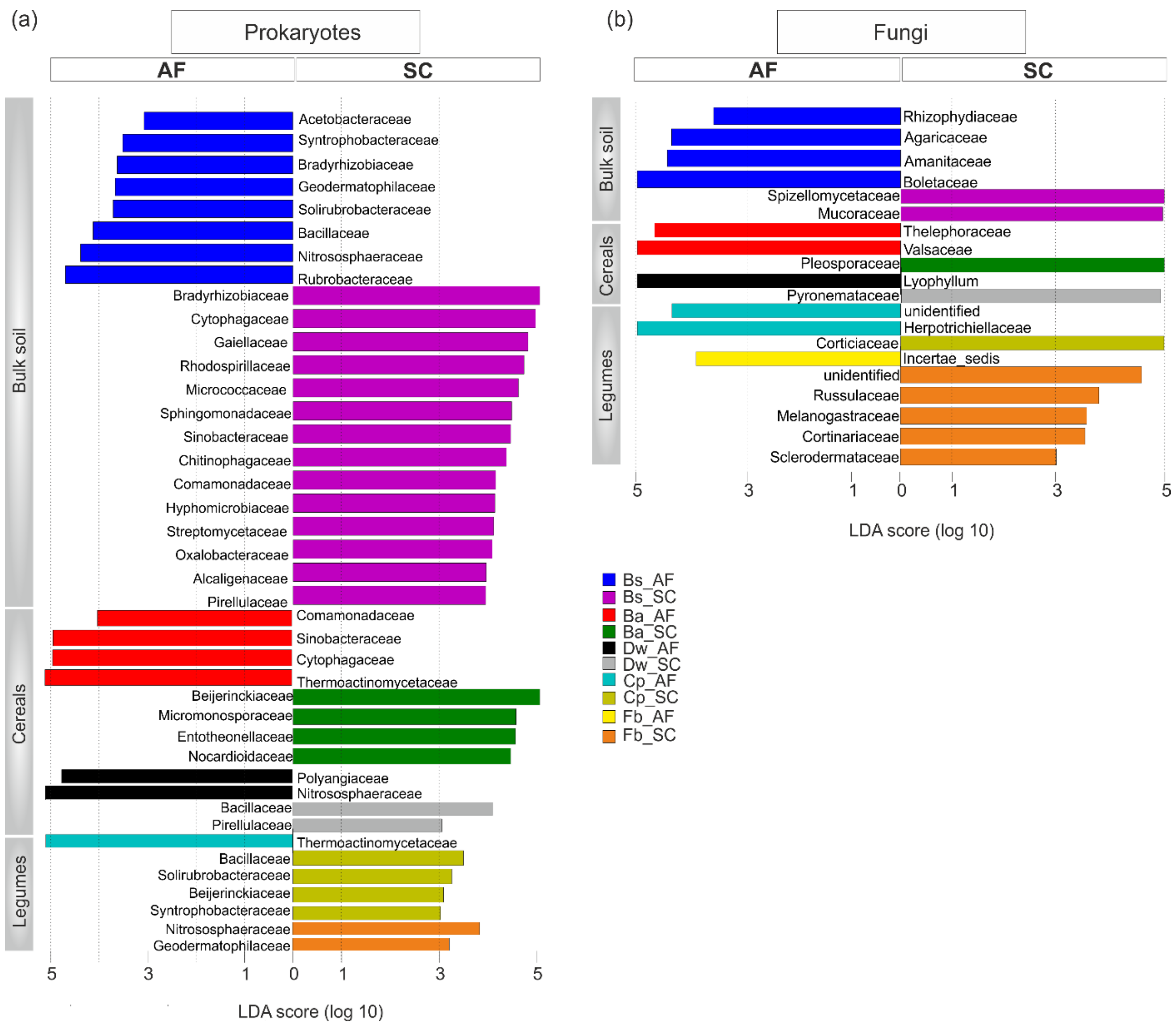

Supplementary Figure S6. Key phylotypes of (a) Prokaryotic and (b) Fungal communities responding to different types of agrosystem identified using LEfSe. LEfSe was used to determine which active taxa have the highest contribution to the observed differences between the two types of agrosystems and the crop species. The histogram shows the LDA scores computed for features (on the OTU level) differentially abundant under the four treatments. Only taxa meeting an LDA significance threshold of 3.0 were shown. AF, agroforestry system; SC, sun cropping; Bs, bulk soil; Ba, barley; Dw, durum wheat; Cp, chickpeas; Fb, Faba bean.

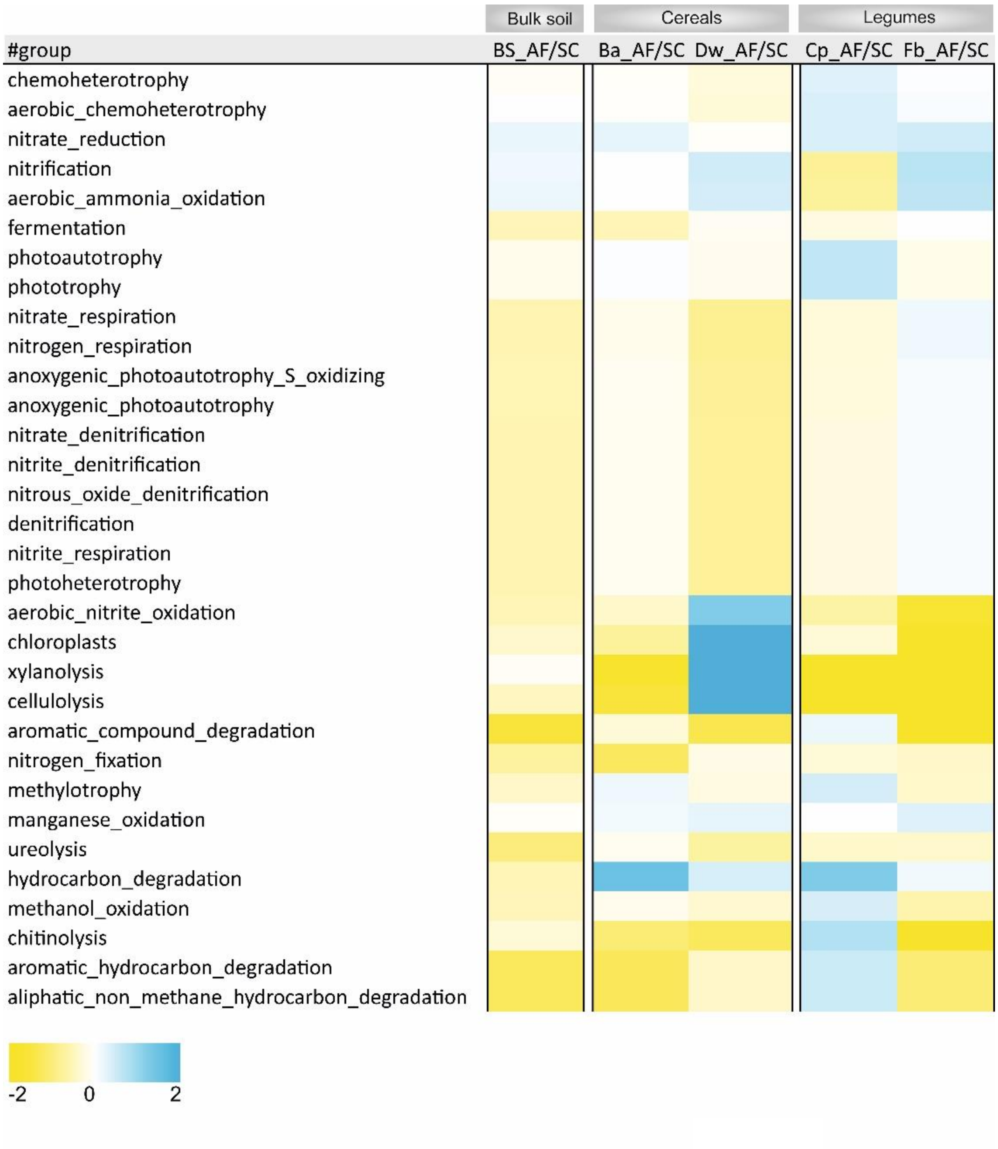

Supplementary Figure S7. Heatmap analysis showing relative abundance of the selected predicted functional potential of the procaryotic rhizospheric community of cereals and legumes using FAPROTAX analysis in the agroforestry system (AC) and sun cropping (SC) systems as compared to their respective bulk soils. The discrepancy of functional group abundances was discussed between AF and SC plots after normalization to their corresponding bulk soil and we explored the functional potential by calculating the 2-fold change of AF/SC ratio. The relative abundance ranges from -2 to 2 and is represented by yellow (-2 – -0.1), white (-0.1- 0.1), and blue (0.1–2), respectively.
